# Regulation of Milk Production by the MAPK/ERK Pathway in Water Buffalo (*Bubalus bubalis)*: Genomic and Molecular Insights

**DOI:** 10.1002/vms3.70703

**Published:** 2025-11-20

**Authors:** Saima Naz, Urwah Ishaque, Ahmad Manan Mustafa Chatha, Babar Maqbool, Qudrat Ullah, Muhammad Farooq, Shabana Naz, Naseer Khan Momand, Ibrahim A. Alhidary

**Affiliations:** ^1^ Department of Zoology Government Sadiq College Women University Bahawalpur Punjab Pakistan; ^2^ Department of Entomology, Faculty of Agriculture and Environment The Islamia University of Bahawalpur Bahawalpur Punjab Pakistan; ^3^ Department of Veterinary Science, Faculty of Animal Husbandry and Veterinary Sciences The University of Agriculture Dera Ismail Khan Pakistan; ^4^ Department of Theriogenology Cholistan University of Veterinary and Animal Sciences Bahawalpur Pakistan; ^5^ Department of soil plant and Food science university of Bari Aldo Moro Bari Italy; ^6^ Department of Zoology Government College Univeristy Faisalabad Pakistan; ^7^ Animal Science Nangarhar University Jalalabad Afghanistan; ^8^ Department of Animal Production College of Food and Agriculture Science King Saud Univeristy Riyadh Saudi Arabia

**Keywords:** genome characterization, lactation regulation, MAPK/ERK pathway genes, milk production, phylogenetic analysis, transcriptional regulation

## Abstract

The MAPK/ERK pathway plays a critical role in the regulation of milk production by controlling cellular processes such as proliferation, differentiation and survival, which are essential for lactogenesis and mammary gland function. *Bubalus bubalis* (Water buffalo), known for its unique physiological and ecological characteristics, serves as an ideal model to explore the evolutionary and molecular roles of MAPK/ERK pathway genes. This study presents the first comprehensive computational analysis of MAPK/ERK genes in *B. bubalis*, identifying 21 key genes involved in the pathway. Phylogenetic analysis clustered these genes into 13 distinct clades, such as MST1, GRB2, RAS, ETS1, JUN and FOS, and revealed close evolutionary relationships with *Bos taurus* and *Camelus bactrianus*. Structural characterization identified 10 conserved motifs, including essential domains like protein kinase, ETS and RAS, reflecting their functional significance. Gene structure analysis revealed substantial variation in exon‐intron patterns, while synteny analysis confirmed collinearity with human orthologs, indicating genomic conservation. Physicochemical analysis highlighted a broad range of molecular weights and isoelectric points, with most proteins classified as hydrophilic and thermostable. Gene duplication and selection analyses revealed seven segmentally duplicated gene pairs, with the JUN‐ETS1 and DUSP6‐MST1 pairs showing evidence of positive selection, suggesting functional divergence. These findings establish a foundational understanding of MAPK/ERK pathway genes in *B. bubalis* and provide valuable insights into potential targets for genetic improvement, selective breeding and sustainable dairy management strategies aimed at enhancing milk production and quality.

## Graphical abstract

Genomic and molecular analyses reveal 21 MAPK/ERK pathway genes in water buffalo, highlighting conserved motifs, gene duplications and positive selection events that underpin lactation regulation. These insights provide potential targets for genetic improvement to enhance milk yield and quality.

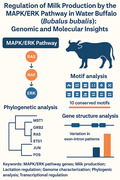

## 1 Introduction

Milk production in mammals is a highly regulated biological process shaped by hormones, cellular signalling and genetic regulation (Al‐Atiyat et al. 2012, 2014; Afriani et al. 2023). In water buffalo (*Bubalus bubalis*), which ranks among the most important contributors to global milk supply, understanding how these regulatory mechanisms work is both scientifically relevant and economically valuable (Maheswarappa et al. 2024). Buffalo milk stands out for its higher fat, protein and mineral content compared to cow milk. This superior composition makes it essential for high‐value dairy products such as cheese and butter across many regions (Khetra et al. 2022). Yet despite its importance, the molecular basis of milk synthesis in buffalo remains poorly described, leaving a gap compared to cattle research.

The mitogen‐activated protein kinase/extracellular signal‐regulated kinase (MAPK/ERK) pathway has emerged as a central regulator of lactation. It controls mammary cell proliferation, differentiation and survival, responding to extracellular signals such as hormones and growth factors. Core components of this cascade include RAS (HRAS, KRAS, NRAS), RAF (BRAF, ARAF), MEK (MAP2K1, MAP2K2) and ERK (MAPK1, MAPK3) (Table S1) (Drosten et al. 2010). Functional studies have shown that activated RAS proteins recruit RAF kinases, which then activate MEK, leading to ERK phosphorylation and nuclear entry (Terrell and Morrison 2019). Inside the nucleus, ERK regulates transcription factors such as FOS, JUN and ELK (Bionaz and Loor 2011), which in bovine mammary cells drive the expression of milk protein genes (Huo et al. 2019). STAT5, a major downstream regulator of this axis, remains essential for casein gene activation and mammary gland development (Tian et al. 2020).

The MAPK/ERK pathway is not linear but tightly balanced. Negative regulators such as dual‐specificity phosphatases (DUSPs), particularly DUSP6, deactivate their components to prevent uncontrolled signalling (Carson et al. 2017). Adaptor proteins like GRB2 and SOS1 assemble pathway complexes and maintain specificity (McDonald et al. 2012). Crosstalk with other cascades further broadens the pathway's role. MST1 and STK3 connect MAPK/ERK with parallel cascades (Nováková et al. 2019). ERK downregulation of PI3K/Akt signalling highlights its ability to integrate survival and growth cues (Hayashi et al. 2008), while reciprocal regulation between MAPK and mTOR pathways has also been confirmed in mammalian cells (Sunayama et al. 2010).

Comparative genomic and transcriptomic work has expanded this understanding. Retinoic acid regulates casein and fatty acid synthesis in bovine mammary epithelial cells (Liao et al. 2020), while microRNAs such as miR‐139 suppress β‐casein synthesis by targeting growth hormone and IGF1 receptor signalling (Cui et al. 2017). These findings point to conserved transcriptional elements across species (Qian and Zhao 1859). In buffalo, Rehman et al. (2021) reported that promoter regions of casein genes contain NF1 and C/EBP binding sites, both influenced by MAPK/ERK signalling. Other studies have linked this pathway with energy homeostasis and metabolic adaptation during lactation (Wu et al. 2022, Cai et al. 2020). These findings point toward buffalo‐specific adaptations, yet the mechanistic basis remains insufficiently understood.

Hormones also act as key modulators of this signalling network. Prolactin activates STAT5 in mammary epithelial cells and drives programs essential for milk synthesis (J. Zhou et al. 2020). Oxytocin, acting through its receptor, activates MAPK/ERK signalling and supports cell differentiation and survival during milk ejection (Jurek and Neumann 2018). IGF‐I contributes to metabolic regulation of lactation by integrating nutrient‐sensing pathways (Wu et al. 2020) and has also been shown to induce COX‐2 expression through MAPK‐dependent signalling (Yuan et al. 2021). Collectively, these hormonal inputs establish MAPK/ERK as a central hub linking endocrine regulation with lactation biology.

Environmental and nutritional inputs further shape MAPK/ERK activity during lactation. Pre‐milking teat stimulation elevates oxytocin release and supports effective milk let‐down in buffalo (B. Li, Khan, et al. 2023). Dietary supplements such as fenugreek enhance milk yield (Terbeche et al. 2023), while methionine activates PI3K–mTOR signalling and promotes protein synthesis (P. Li, Fang, et al. 2023). Stress conditions such as mastitis trigger MAPK cascades (Guo et al. 2019), and DUSP6 acts as a regulator by limiting excessive inflammatory responses (Carson et al. 2017).

Beyond physiological regulation, genomic studies also provide support for MAPK/ERK's role in lactation. NF1‐C2 polymorphisms in goats have been associated with milk yield (P. Wang et al. 2011), while GWAS in cattle identified MAPK/ERK‐related regulators (Pegolo et al. 2018). In buffalo, PPARGC1A has been highlighted as a key gene controlling milk fat synthesis (Qiu et al. 2020). Together, these studies highlight conserved MAPK/ERK functions across ruminants but underscore the lack of buffalo‐specific analyses.

This study therefore presents the first comprehensive computational analysis of MAPK/ERK pathway genes in buffalo, integrating phylogenetic, structural, synteny and transcription factor binding analyses. These insights not only advance buffalo lactation biology but also provide molecular tools for selective breeding and sustainable dairy management.

## 2 Results

### 2.1 Phylogenetic Relationships in the MAPK/ERK Pathway Genes

**FIGURE 1:**
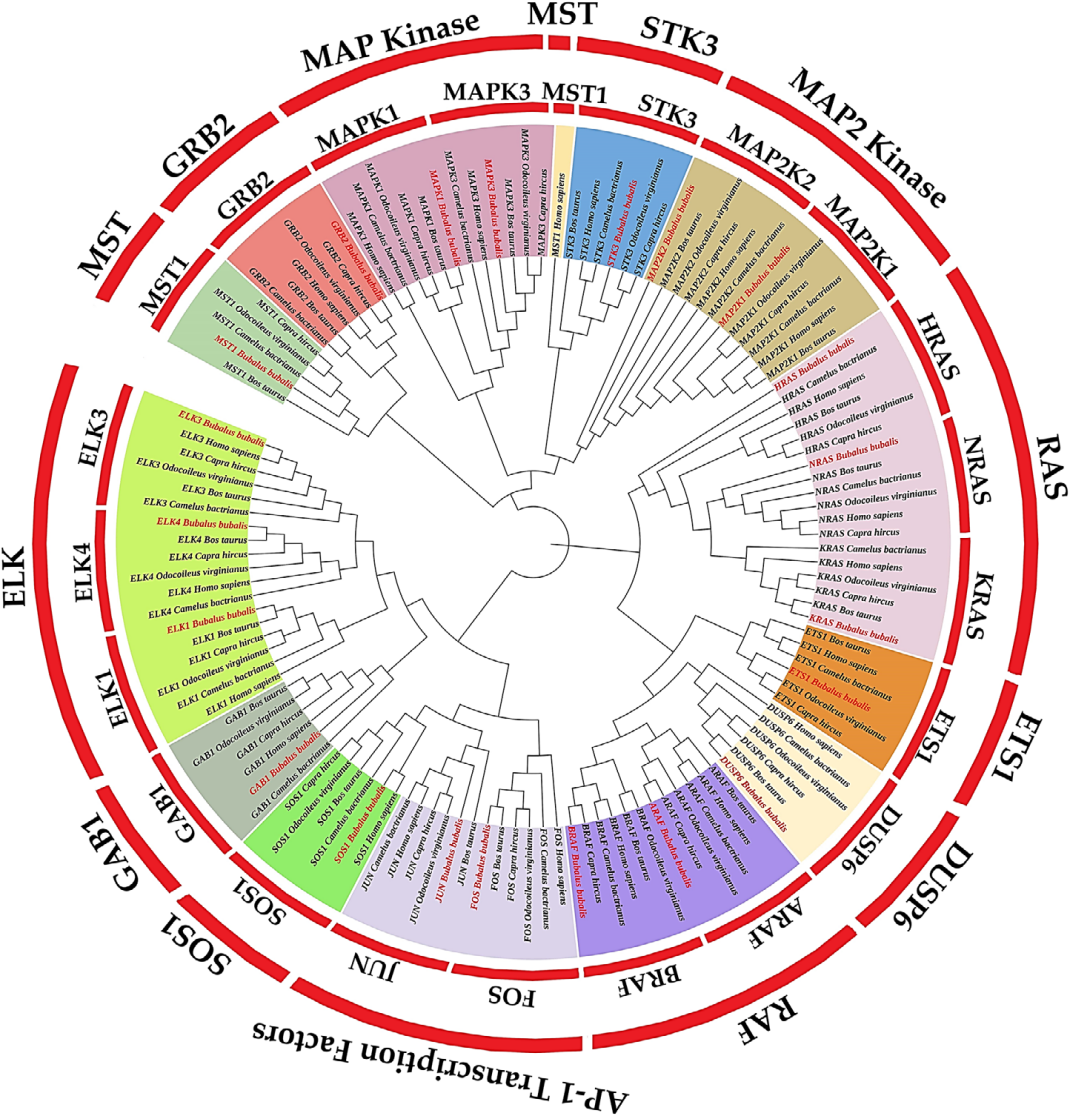
Maximum‐likelihood phylogenetic tree of MAPK/ERK pathway genes in *B. bubalis* with reference species (*B. taurus*, *C. bactrianus*, *O. virginianus*, *C. hircus* and *H. sapiens*). The tree grouped the genes into thirteen major clades: MST1, GRB2, MAP kinase, STK3, MAP2 kinase, RAS, ETS1, DUSP6, RAF, AP‐1 transcription factors (FOS, JUN), SOS1, GAB1 and ELK. Coloured branches show a close similarity between buffalo genes and those of cattle and camel, pointing to the strong evolutionary conservation of this pathway across ruminants.

The phylogenetic analysis of 126 MAPK/ERK pathway protein sequences from buffalo and five reference species grouped them into 13 major clades that represent the core signalling components (Figure 1). Buffalo genes clustered most closely with cattle and camel orthologs, which is expected given their close evolutionary relationship. JUN and ETS1 lined up tightly with bovine counterparts, supporting their conserved role in regulating milk protein synthesis.

An interesting exception came with human MST1, which fell into the STK3 clade instead of grouping with other MST1 orthologs. This shift hints at lineage‐specific variation in primates, while buffalo MST1 kept its conserved placement alongside livestock species.

### 2.2 Characterization of the Structure of MAPK/ERK Pathway Genes

**FIGURE 2:**
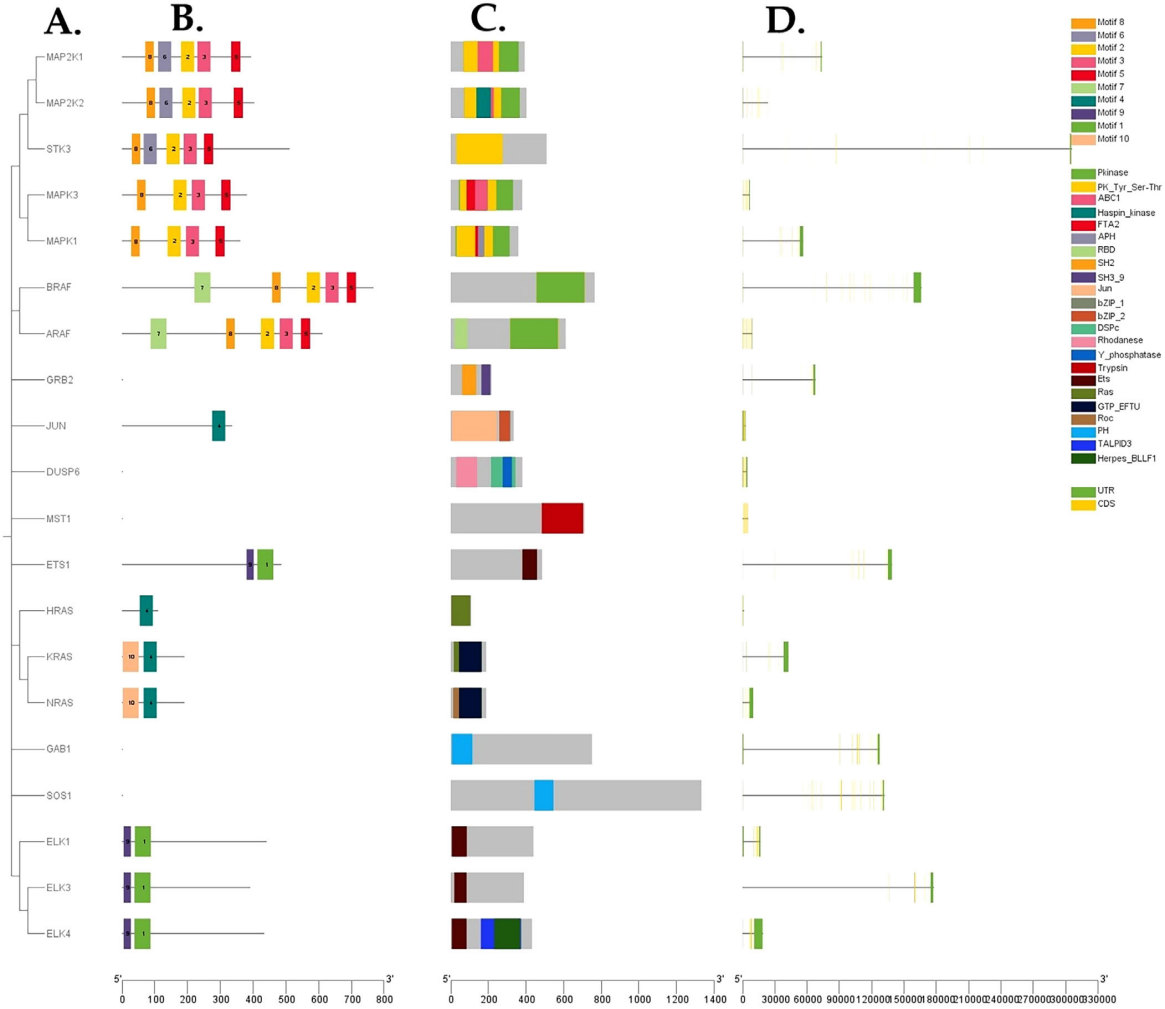
Conserved motifs, domains and gene structures of buffalo MAPK/ERK genes. (A) Phylogenetic tree showing the relationships among *B. bubalis* MAPK/ERK genes. (B) Distribution of 10 conserved motifs identified through MEME, each coloured box representing a specific motif with its number displayed above. (C) Functional domains confirmed by InterPro and NCBI CDD, including Protein Kinase, ETS, RAS and ZF‐DAG. (D) Exon–intron organization extracted from GTF files, where UTRs are shown in grey, exons in green and introns as black lines.

Structural analysis of buffalo MAPK/ERK genes showed conserved motifs and domains that define this signalling cascade (Figure 2). MEME detected 10 conserved motifs across 16 genes, with several (MEME‐1, MEME‐7, MEME‐10) linked to core protein kinase or transcription factor regions. A few genes, including GRB2, DUSP6, MST1, GAB1 and SOS1, did not contain these motifs, pointing toward functional divergence inside the pathway. InterPro and CDD annotations confirmed domains such as Protein Kinase, ETS, RAS and ZF‐DAG, underlining their conservation across species (Table 1; Figure 2B,C).

**TABLE 1:** A total of 10 highly conserved motifs identified within the MAPK/ERK pathway genes of *B. bubalis*, detailing amino acid sequences, motif widths and associated domains.

MEME Motif	Amino acid sequence	Width	InterPro Domain
1	AEEVARLWGLRKNKPNMNYDKLSRALRYYYDKNIIKKVAGQKFVYKFVSY	50	ETS domain
2	YJHAKNIIHRDLKPSNILLNTRGEIKJCDFGLAGVLTDSMA	41	Protein kinase domain
3	MPPEMLQSTGYSVQSDIWSVGIVLVEMLTGRPPYPGIHYKD	41	Protein kinase domain
4	AMRDQYMRTGEGFLCVFAINNTKSFEDIHQYREQIKRVKDS	41	RAS domain
5	DFQDLLNKCLIKNPEERADLEQLLAHPYL	29	Protein kinase domain
6	QIIRELQVLHECNSPYIVGFYGAFYSDGEISICMEHMDGGS	41	Protein kinase domain
7	IVEVLEDVPLTMHNFVRKTFFSLAFCDFCRKFLFQGFRCQTCGYKFHQRC	50	ZF‐DAG domain
8	LSRJGEGSYGTVFKAYHHPSGAVVAJK	27	Protein kinase domain
9	ITLWQFLLQLLKDQKNEHLISWT	23	ETS domain
10	TEYKLVVVGAGGVGKSALTIQLIQNHFVDEYDPTIEDSYRKQVVIDGETC	50	RAS domain

Gene structure analysis further revealed variation in exon–intron length and UTR arrangement (Figure 2D). These shifts may add regulatory flexibility, allowing differences in transcriptional control and possibly contributing to adaptive roles of MAPK/ERK signalling in buffalo.

### 2.3 Physicochemical Analysis of MAPK/ERK Pathway Genes

Physicochemical profiling showed clear variation in protein size, charge and stability among buffalo MAPK/ERK members (Table 2). Most proteins carried acidic isoelectric points (pIs), while ARAF, ELK3 and JUN shifted toward basic ranges, hinting at differences in their subcellular interactions. The instability index (II) classified most proteins as unstable, but HRAS and NRAS came out relatively stable, pointing to stronger functional robustness within the RAS family.

**TABLE 2:** Physicochemical properties of the MAPK/ERK pathway genes in *B. bubalis*.

Gene name	Chr. No	Exon count	Molecular weight (D)	Amino acid	Isoelectric point	Instability index	Aliphatic index	GRAVY
BRAF	8	23	84,506.94	765	7.27	53.81	77.65	−0.38
ARAF	X	17	68,117.8	611	9.28	50.26	76.42	−0.381
MAP2K1	11	14	43,439.01	393	6.18	43.85	87.07	−0.305
MAP2K2	9	13	44,816.49	403	5.89	54.4	89.53	−0.357
MAPK3	24	9	43,115.48	380	6.03	39.46	92.16	−0.308
MAPK1	17	10	41,375.68	360	6.5	40.24	95.67	−0.287
HRAS	9	3	12,357.77	109	4.73	33.42	73.21	−0.509
KRAS	4	7	21,641.8	189	6.33	38.11	84.5	−0.433
NRAS	6	7	21,197.11	189	5.01	28.98	84.5	−0.305
GRB2	3	6	25,206.35	217	5.89	37.93	65.58	−0.668
FOS	11	4	40,764.42	380	4.77	76.84	62.24	−0.438
JUN	6	1	36,083.95	335	8.9	55.25	76.09	−0.476
DUSP6	4	3	42,333.77	381	4.75	51.54	86.22	−0.263
ELK1	X	6	46,240.45	440	5.81	60.3	77.82	−0.32
ELK3	4	9	42,744.01	389	9.12	68.19	91.31	−0.29
ELK4	5	6	46,733.16	432	6.93	57.21	76.99	−0.417
MST1	21	18	80,221.96	711	7.76	45.31	63.88	−0.553
STK3	15	16	58,302.24	510	5.29	55.2	72.1	−0.644
SOS1	12	23	152,407.98	1333	6.34	61.45	81.76	−0.545
GAB1	17	15	82,796.08	751	5.62	64.39	61.53	−0.765
ETS1	5	13	55,322.07	486	5	53.45	65.64	−0.562

Hydropathy analysis gave mostly negative GRAVY values, showing that the majority of proteins are hydrophilic. GRB2 and GAB1 stood out as highly hydrophilic, which fits with their adaptor roles in assembling complexes at the membrane. Aliphatic index (AI) values suggested overall thermostability across the set, supporting their ability to stay functional under physiological stress.

### 2.4 Collinearity Analysis

**FIGURE 3:**
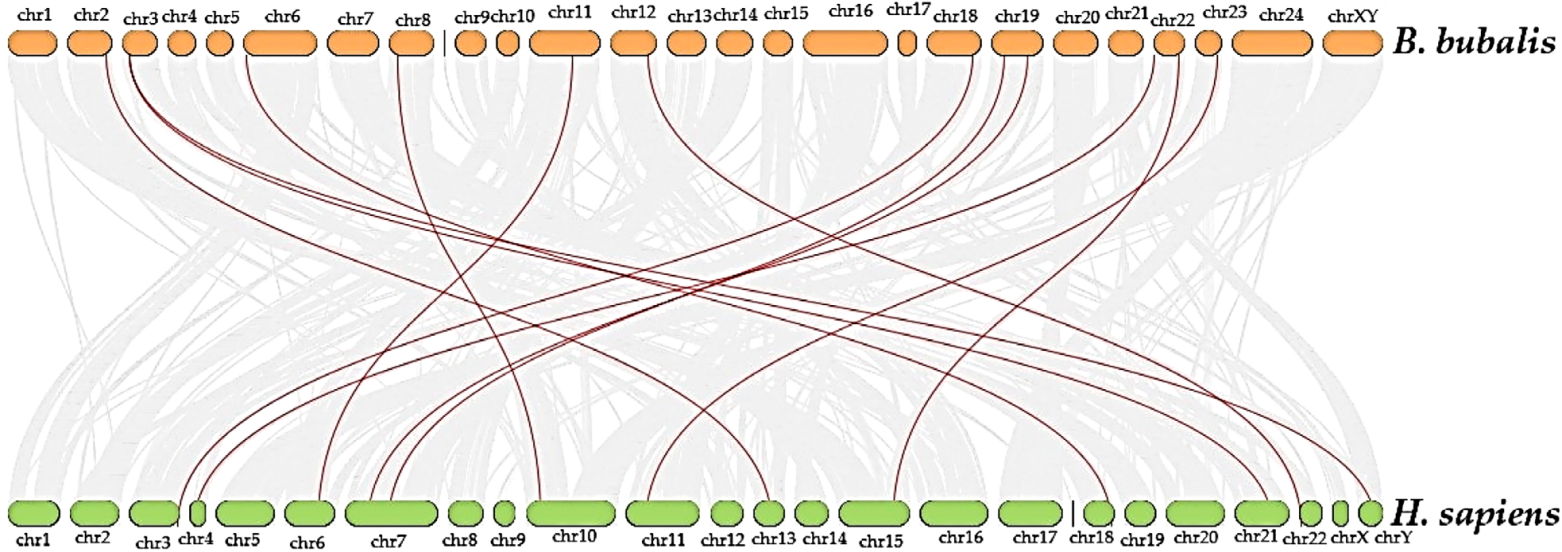
Linear synteny map of MAPK/ERK pathway genes between *B. bubalis* (top; orange chromosomes chr1–chr24, chrX/Y) and *H. sapiens* (bottom; green chromosomes chr1–chr22, chrX/Y). Each rounded block represents a chromosome, with IDs labelled above or below. Red ribbons connect orthologous MAPK/ERK loci, while thin grey ribbons in the background mark genome‐wide collinear blocks for comparison. This layout shows how MAPK/ERK genes are conserved across the two species and underlines the large‐scale collinearity that shaped their genomic architecture.

Collinearity analysis showed strong syntenic conservation of MAPK/ERK pathway genes between buffalo and human genomes (Figure 3). Most genes were positioned near chromosomal terminal regions, a pattern that could support recombination and add to regulatory diversity. Conserved collinear blocks pointed to overall genomic stability, while syntenic MAPK/ERK gene pairs underlined functional conservation across species.

Together, these results suggest that even with evolutionary divergence, the genomic organization of MAPK/ERK components has stayed largely intact. This highlights their essential role in signalling and points toward regulatory importance in buffalo lactation.

### 2.5 Duplication and Localization of MAPK/ERK Pathway Genes

Analysis of buffalo MAPK/ERK pathway genes showed that all seven duplication events were segmental, confirming their role in gene family expansion (Table 3). Most gene pairs, including MAPK3–MAPK1 and MAP2K1–MAP2K2, carried Ka/Ks ratios below 1, which points to purifying selection and functional conservation. In contrast, JUN–ETS1 and DUSP6–MST1 showed ratios above 1, suggesting positive selection and adaptive divergence. This pattern indicates that transcriptional regulators like JUN and ETS1, along with signalling modulators such as DUSP6 and MST1, may have developed specialized roles in lactation biology.

**TABLE 3:** Evaluation of Ka/Ks ratios for gene pairs in the MAPK/ERK pathway of *B. bubalis*.

Gene Pairs	Chromosomes	Duplication	Ka	Ks	Ka/Ks	Selection	Time (MYA)
ELK3‐ELK4	4/5	SD	0.7075	—	—	—	—
JUN‐ETS1	6/5	SD	1.8603	0.8044	2.312655	Positive selection	36.56
MAPK3‐MAPK1	24/17	SD	0.0591	0.6617	0.089315	Purifying selection	30.08
MAP2K1‐MAP2K2	11/9	SD	0.1137	0.7771	0.146313	Purifying selection	35.32
DUSP6‐MST1	4/21	SD	2.0714	1.1928	1.736586	Positive selection	54.22
BRAF‐ARAF	8/X	SD	0.4733	1.5011	0.315302	Purifying Selection	68.23
KRAS‐NRAS	4/6	SD	0.0296	2.3482	0.012605	Purifying Selection	106.74

Abbreviations: Ka, non‐Synonymous substitutions; Ks, synonymous substitutions; MYA, millions of years ago; SD, Segmental duplication.

Duplication timing added further insight. Older events such as KRAS–NRAS (∼107 Mya) reflected stable, conserved functions within the RAS family, while more recent events like JUN–ETS1 (∼37 Mya) suggested lineage‐specific adaptations that may influence milk protein regulation in buffalo.

**FIGURE 4:**
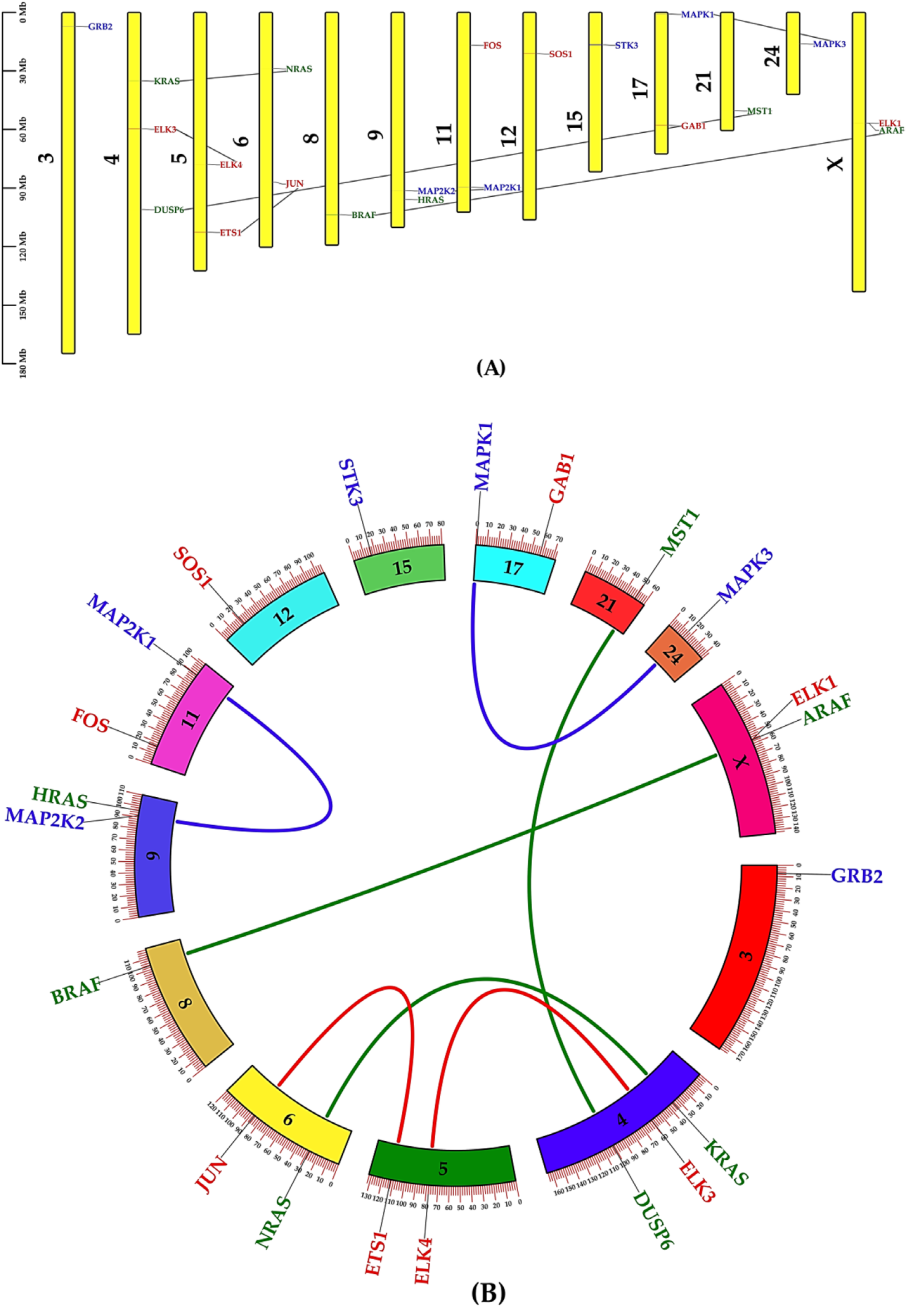
Chromosomal mapping and gene duplication of buffalo MAPK/ERK genes. (A) Chromosomal distribution across *B. bubalis* with gene names marked at each position, while grey links represent pairs under purifying selection. (B) Circular plot showing seven segmentally duplicated gene pairs. Colored links connect duplications under positive selection (JUN–ETS1 and DUSP6–MST1). Together, these results highlight how segmental duplications shaped the evolutionary history of the pathway.

Chromosomal mapping (Figure 4) confirmed segmental duplications spread across multiple chromosomes, underscoring the structural complexity of MAPK/ERK organization in buffalo and pointing to its evolutionary diversification.

### 2.6 Prediction of Secondary and Tertiary Protein Structures

**FIGURE 5:**
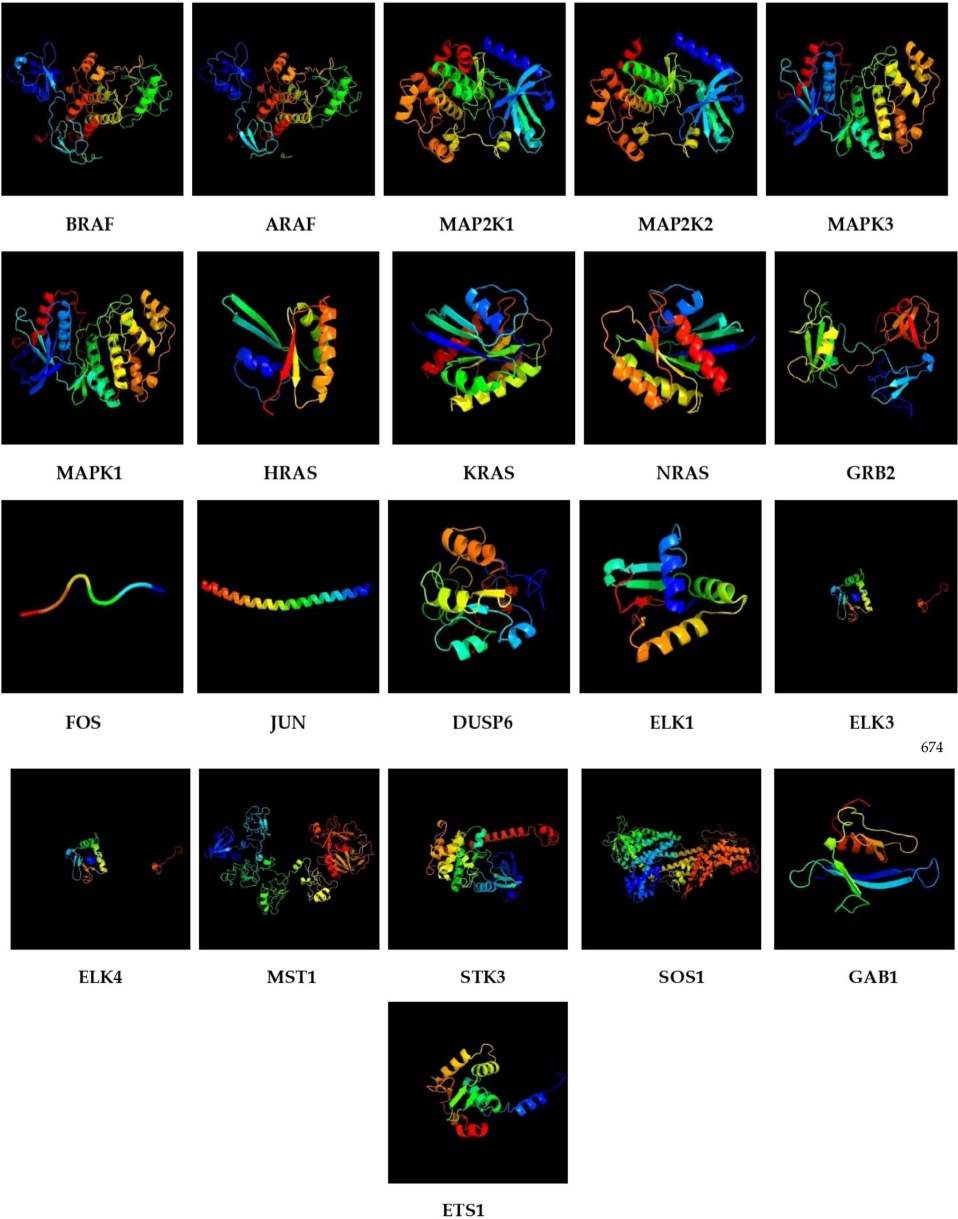
Predicted tertiary structures of MAPK/ERK pathway proteins in *B. bubalis*. Homology models for BRAF, ARAF, MAP2K1, MAP2K2, MAPK3, MAPK1, HRAS, KRAS, NRAS, GRB2, FOS, JUN, DUSP6, ELK1, ELK3, ELK4, MST1, STK3, SOS1, GAB1 and ETS1 are shown, with each ribbon model coloured from N‐ to C‐terminus to indicate residue order. These models illustrate the canonical folds of kinases, small GTPases, adaptor proteins and transcription factors involved in the buffalo MAPK/ERK pathway. Extended segments likely reflect predicted intrinsically disordered regions.

Structural predictions showed that buffalo MAPK/ERK proteins share conserved folding patterns but differ in their levels of intrinsic disorder (Table 4; Figure 5, Figures S1–S2). Many proteins carried large disordered regions, which likely support flexible protein–protein interactions common in signalling cascades. In contrast, proteins such as MAP2K1 and SOS1 contained higher proportions of alpha helices, pointing to a more stable structural organization.

**TABLE 4:** Predicted secondary structure characteristics of the twenty‐one MAPK/ERK pathway genes in *B. bubalis* using Phyre2.

Sr. No	Name of Protein	Alpha Helix 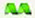	Beta strand 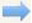	TM strand 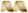	Disorder 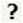
1	BRAF	22%	9%	0%	43%
2	ARAF	18%	13%	3%	36%
3	MAP2K1	32%	12%	4%	23%
4	MAP2K2	28%	13%	4%	25%
5	MAPK3	26%	14%	4%	12%
6	MAPK1	26%	16%	4%	9%
7	HRAS	16%	43%	0%	1%
8	KRAS	33%	29%	0%	12%
9	NRAS	33%	28%	0%	11%
10	GRB2	2%	38%	0%	17%
11	FOS	17%	1%	0%	85%
12	JUN	24%	0%	0%	84%
13	DUSP6	26%	9%	0%	24%
14	ELK1	8%	4%	0%	80%
15	ELK3	11%	6%	0%	74%
16	ELK4	8%	5%	0%	79%
17	MST1	5%	26%	0%	3%
18	STK3	27%	10%	0%	49%
19	SOS1	39%	4%	0%	36%
20	GAB1	4%	7%	0%	84%
21	ETS1	18%	5%	0%	61%

Abbreviations: TM, transmembrane helix.

Three‐dimensional models built through Phyre2 and validated by AlphaFold2 produced high confidence scores, confirming the structural reliability of these proteins. Overall, the results suggest a balance between stability and disorder in buffalo MAPK/ERK proteins, a feature that may underlie their adaptability in signal transduction and their roles in lactation‐related regulation.

### 2.7 Scan PROSITE Analysis

**FIGURE 6:**
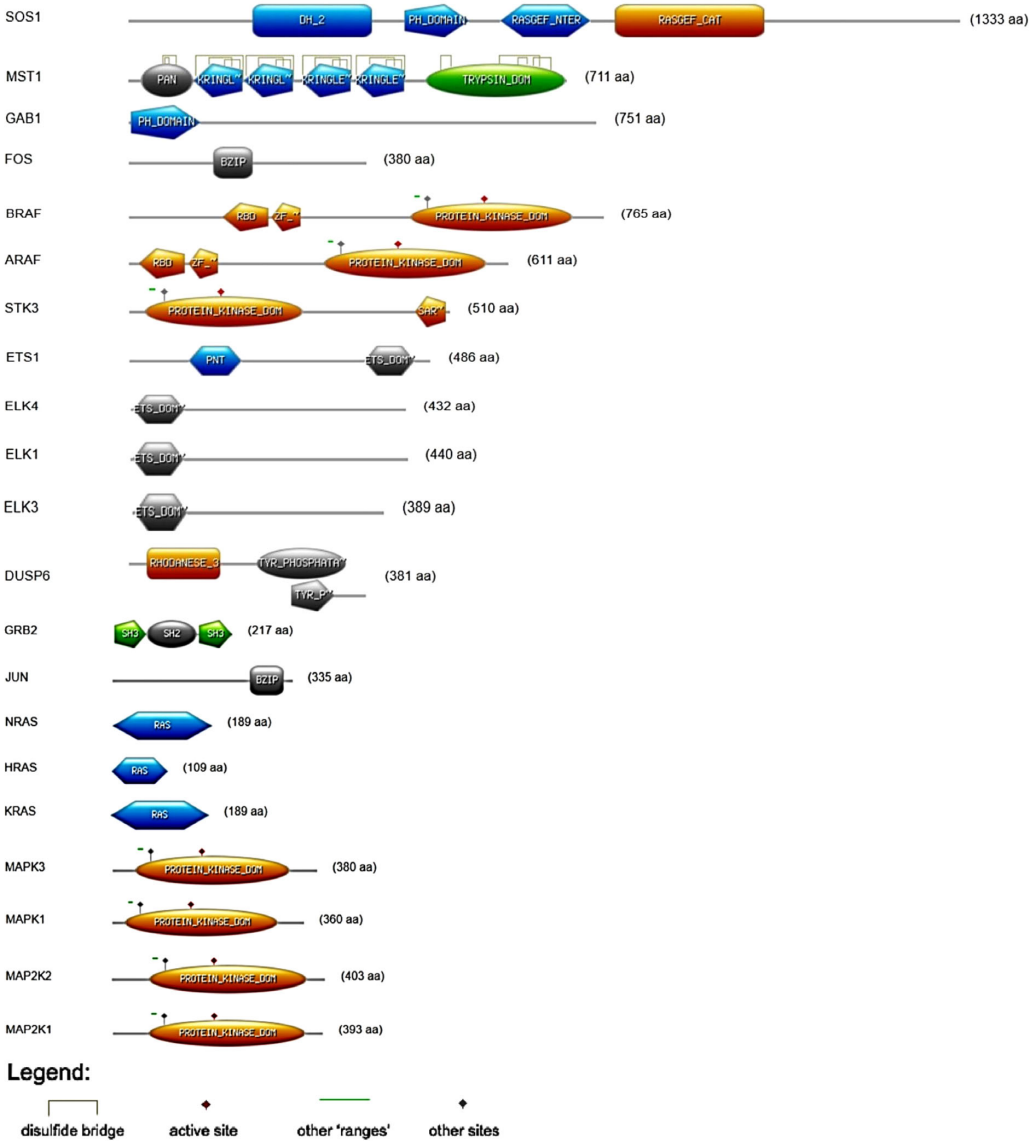
Domain architecture and functional motifs of buffalo MAPK/ERK proteins identified with ScanPROSITE. Schematics display kinase, ETS, Ras, PH and bZIP domains, as well as active sites and disulphide bridges. Colour coding marks enzymatic activity, DNA binding and protein–protein interactions. This variation reflects the biochemical diversity of MAPK/ERK proteins and their potential regulatory impact on milk production.

Domain analysis showed that buffalo MAPK/ERK proteins retain the core features needed for signal transduction (Figure 6). Kinase domains in MAPK1, MAPK3 and BRAF confirmed their enzymatic role, while Ras domains in NRAS, HRAS and KRAS reflected conserved GTPase‐mediated signalling. Transcription factors such as ELK, ETS, FOS and JUN carried DNA‐binding domains (ETS, bZIP), supporting their role in regulating gene expression.

Adaptor proteins, including SOS1 and GAB1, contained interaction motifs such as PH domains, which highlight their role in assembling signalling complexes. The presence of disulphide bridges and active sites further emphasized the biochemical flexibility of the pathway. This domain architecture shows how buffalo MAPK/ERK proteins combine enzymatic activity, transcriptional control and regulatory interactions to sustain robust signalling during lactation.

### 2.8 Transcription Factor Binding Sites Analysis

Analysis of buffalo MAPK/ERK pathway genes revealed extensive regulation by key transcription factors (Table 5). STAT5A accounted for the largest number of predicted binding sites, highlighting its central role in activating casein gene expression and mammary gland development. cMyc and GATA‐3 were also widely represented, consistent with their functions in cell proliferation and differentiation. NF‐κB sites, though fewer, suggest an additional layer of regulation under immune or stress‐related conditions. These results indicate that buffalo MAPK/ERK pathway genes are embedded within a complex transcriptional network, integrating lactation, growth and immune responses.

**TABLE 5:** Transcription factor binding sites (TFBSs) for genes in the MAPK/ERK pathway in *B. bubalis*, showing the specific TFBSs (STAT‐5A, GATA‐3, cMyc and NF‐kB) analysed in this study, along with the total number of binding sites, associated amino acid sequences and their respective locations within each gene.

Gene name	TFBSs	Total Sites	Amino acid sequence	Amino acid localization
BRAF	STAT‐5A	21	GGAA, TTCC, TTCC, TTCC, TTCC, GGAA, TTCC, TTCC, TTCC, TTCC, TTCC, GGAA, G GAA, GGAA, TTCC, TTCC, TTCC, TTCC, GGAA, GGAA, TTCC	15–18, 41–44, 48–51, 63–66, 263–266, 296–299, 474–477, 591–594, 609–612, 622–625, 676–679, 813–816, 888–891, 927–930, 957–960, 1075–1078, 1197–1200, 1204–1207, 1306–1309, 1324–1327, 1463–1466
	GATA‐3	1	TTCTATCT	429–436
	cMyc	1	TCAGGTG	1412–1418
	NF‐kB	3	CTGGCTTTTCC, ATGGGCTTTCC, GGAAACGGCCG	1068–1078, 1190–1200, 1306–1316
ARAF	STAT‐5A	23	TTCC, GGAA, TTCC, GGAA, TTCC, GGAA, GGAA, TTCC, TTCC, GGAA, GGAA, GGAA, TTCC, TTCC, TTCC, TTCC, GGAA, GGAA, TTCC, GGAA, GGAA, TTCC, TTCC	35–38, 125–128, 255–258, 273–276, 294–297, 353–356, 663–666, 725–728, 808–811, 913–916, 932–935, 939–942, 1016–1019, 1035–1038, 1049–1052, 1098–1101, 1106–1109, 1110–1113, 1244–1247, 1281–1284, 1349–1352, 1401–1404, 1448–1451
	GATA‐3	4	AGATACAA, ACATATCT, AGATAGGG, CGCTATCT	529–536, 676–683, 740–747, 748–755
	cMyc	2	CACATGC, CCAAGTG	1009–1015, 1368–1374
	NF‐kB	2	GGAAAGTCTCA, AGGTCTTTTCC	939–949, 1441–1451
MAP2K1	STAT‐5A	18	TTCC, TTCC, GGAA, GGAA, GGAA, GGAA, TTCC, GGAA, GGAA, GGAA, TTCC, TTCC, TTCC, GGAA, TTCC, TTCC, GGAA, GGAA	36–39, 59–62, 223–226, 280–283, 391–394, 535–538, 552–555, 798–801, 805–808, 903–906, 950–953, 1009–1012, 1115–1118, 1140–1143, 1293–1296, 1394–1397, 1471–1474, 1488–1491
	GATA‐3	4	ATTTATCT, AAATATCT, AATTATCT, AGATATAT	86–93, 557–564, 905–912, 943–950
	cMyc	2	CACCTGG, CCAGGTG	1306–1312, 1457–1463
	NF‐kB	1	GGAAAATCCCA	280–290
MAP2K2	STAT‐5A	17	GGAA, TTCC, GGAA, TTCC, GGAA, TTCC, GGAA, GGAA, TTCC, GGAA, GGAA, GGAA, GGAA, GGAA, GGAA, TTCC, TTCC	41–44, 54–57, 160–163, 203–206, 273–276, 436–439, 474–477, 601–604, 737–740, 778–781, 801–804, 917–920, 963–966, 1117–1120, 1248–1251, 1337–1340, 1430–1433
	GATA‐3	2	AGATATCC, AGATATTG	450–457, 485–492
	cMyc	4	CACATGC, CACATGC, CACATGA, CACCTGC	286–292, 360–366, 421–427, 1290–1296
	NF‐kB	1	AGGGACTTCCC	197–207
MAPK3	STAT‐5A	16	TTCC, GGAA, TTCC, TTCC, GGAA, TTCC, TTCC, TTCC, TTCC, TTCC, TTCC, TTCC, GGAA, TTCC, GGAA, GGAA	9–12, 133–136, 221–224, 270–273, 276–279, 492–495, 521–524, 739–742, 850–853, 928–931, 994–997, 1019–1022, 1051–1054, 1121–1124, 1366–1369, 1466–1469
	GATA‐3	2	AGATACAT, GATTATCT	456–463, 880–887
	cMyc	7	CACATGT, ACATGTG, CCACGTG, CACGTGG, ACAGGTG, TCACGTG, CACGTGA	1040–1046, 1041–1047, 1171–1177, 1172–1178, 1335–1341, 1395–1401, 1396–1402
	NF‐kB	1	CCGGGCTTTCC	987–997
MAPK1	STAT‐5A	12	TTCC, TTCC, TTCC, GGAA, GGAA, GGAA, TTCC, GGAA, GGAA, TTCC, TTCC, TTCC	86–89, 337–340, 422–425, 561–564, 681–684, 824–827, 889–892, 950–953, 993–996, 1120–1123, 1219–1222, 1361–1364
	GATA‐3	—	—	—
	cMyc	1	TCATGTG	379–385
	NF‐kB	1	AGGTTCTTTCC	79–89
HRAS	STAT‐5A	14	TTCC, GGAA, TTCC, TTCC, TTCC, TTCC, TTCC, GGAA, GGAA, TTCC, GGAA, TTCC, GGAA, TTCC	33–36, 52–55, 175–178, 428–431, 471–474, 556–559, 691–694, 719–722, 897–900, 1029–1032, 1143–1146, 1253–1256, 1413–1416, 1421–1424
	GATA‐3	—	—	—
	cMyc	5	ACAAGTG, CACCTGT, CACCTGC, TCAGGTG, GCAGGTG	180–186, 490–496, 506–512, 880–886, 998–1004
	NF‐kB	—	—	—
KRAS	STAT‐5A	9	TTCC, TTCC, GGAA, TTCC, GGAA, TTCC, TTCC, TTCC, TTCC	63–66, 138–141, 341–344, 588–591, 980–983, 1000–1003, 1365–1368, 1472–1475, 1479–1482
	GATA‐3	3	AGATATTC, AGATAGTT, GTATATCT	206–213, 538–545, 739–746
	cMyc	2	TCAGGTG, CCAAGTG	725–731, 1082–1088
	NF‐kB	1	TGGGATTTTCC	56–66
NRAS	STAT‐5A	11	GGAA, TTCC, GGAA, TTCC, TTCC, TTCC, TTCC, TTCC, GGAA, TTCC, GGAA	244–247, 286–289, 358–361, 576–579, 665–668, 770–773, 793–796, 1057–1060, 1124–1127, 1255–1258, 1364–1367
	GATA‐3	4	GAATATCT, AATTATCT, TTTTATCT, ATTTATCT	212–219, 763–770, 1248–1255, 1302–1309
	cMyc	5	CACATGG, CACTTGA, CACTTGA, CCACGTG, CACGTGG	61–67, 500–506, 1082–1088, 1487–1493, 1488–1494
	NF‐kB	—	—	—
GRB2	STAT‐5A	21	TTCC, TTCC, GGAA, GGAA, GGAA, TTCC, TTCC, GGAA, TTCC, TTCC, TTCC, TTCC, GGAA, TTCC, TTCC, TTCC, TTCC, GGAA, GGAA, TTCC, GGAA	98–101, 312–315, 317–320, 372–375, 454–457, 477–480, 498–501, 510–513, 641–644, 707–710, 729–732, 906–909, 928–931, 967–970, 972–975, 986–989, 1150–1153, 1228–1231, 1234–1237, 1446–1449, 1456–1459
	GATA‐3	—	—	—
	cMyc	2	CACTTGA, CACCTGC	801–807, 1349–1355
	NF‐kB	1	AGGCCTTTTCC	1439–1449
FOS	STAT‐5A	16	TTCC, TTCC, TTCC, GGAA, TTCC, TTCC, GGAA, TTCC, TTCC, TTCC, TTCC, GGAA, TTCC, TTCC, GGAA, GGAA	190–193, 233–236, 263–266, 312–315, 347–350, 764–767, 776–779, 835–838, 889–892, 964–967, 973–976, 1033–1036, 1161–1164, 1226–1229, 1302–1305, 1351–1354
	GATA‐3	1	AGATAAAG	726–733
	cMyc	2	CCAAGTG, CCAGGTG	398–404, 1054–1060
	NF‐kB	1	GGAAAATCACC	312–322
JUN	STAT‐5A	21	GGAA, GGAA, TTCC, TTCC, GGAA, TTCC, GGAA, TTCC, GGAA, TTCC, TTCC, TTCC, TTCC, GGAA, GGAA, TTCC, TTCC, TTCC, GGAA, GGAA, GGAA	16–19, 126–129, 187–190, 206–209, 274–277, 299–302, 354–357, 420–423, 425–428, 540–543, 556–559, 622–625, 738–741, 904–907, 1004–1007, 1051–1054, 1086–1089, 1116–1119, 1221–1224, 1246–1249, 1439–1442
	GATA‐3	1	AGATAAGT	1490–1497
	cMyc	5	CACTTGC, GCAAGTG, ACATGTG, CCAAGTG, CACCTGG	103–109, 392–398, 477–483, 996–1002, 1039–1045
	NF‐kB	1	GGAAATACCAC	1004–1014
DUSP6	STAT‐5A	20	GGAA, TTCC, TTCC, GGAA, GGAA, TTCC, GGAA, GGAA, TTCC, TTCC, TTCC, GGAA, GGAA, TTCC, GGAA, TTCC, GGAA, TTCC, TTCC, TTCC	22–25, 28–31, 177–180, 430–433, 434–437, 555–558, 652–655, 749–752, 816–819, 881–884, 897–900, 1161–1164, 1171–1174, 1271–1274, 1302–1305, 1378–1381, 1433–1436, 1438–1441, 1443–1446, 1493–1496
	GATA‐3	1	TATTATCT	885–892
	cMyc	1	CACTTGA	516–522
	NF‐kB	1	GGAAATTCCTT	1433–1443
ELK1	STAT‐5A	14	GGAA, TTCC, TTCC, GGAA, TTCC, TTCC, GGAA, TTCC, GGAA, TTCC, GGAA, TTCC, TTCC, TTCC	59–62, 162–165, 168–171, 488–491, 680–683, 716–719, 893–896, 1065–1068, 1128–1131, 1142–1145, 1185–1188, 1291–1294, 1316–1319, 1373–1376
	GATA‐3	2	TGCTATCT, CTCTATCT	752–759, 1073–1080
	cMyc	3	CACCTGG, CCAGGTG, CACATGT	137–143, 438–444, 1031–1037
	NF‐kB	1	GCAGAATTTCC	1284–1294
ELK3	STAT‐5A	24	TTCC, GGAA, GGAA, GGAA, TTCC, GGAA, TTCC, TTCC, GGAA, TTCC, GGAA, GGAA, GGAA, TTCC, TTCC, TTCC, TTCC, GGAA, TTCC, TTCC, TTCC, TTCC, TTCC, TTCC	38–41, 163–166, 190–193, 213–216, 287–290, 300–303, 306–309, 354–357, 366–369, 443–446, 526–529, 648–651, 883–886, 933–936, 943–946, 966–969, 987–990, 1030–1033, 1191–1194, 1212–1215, 1240–1243, 1333–1336, 1341–1344, 1449–1452
	GATA‐3	3	GGTTATCT, ATATATCT, AGATAGTA	449–456, 727–734, 1197–1204
	cMyc	4	ACATGTG, GCAGGTG, CACCTGC, CACATGG	166–172, 390–396, 899–905, 1001–1007
	NF‐kB	2	TGGAATTTTCC, GGAGATTTTCC	299–309, 436–446
ELK4	STAT‐5A	20	TTCC, GGAA, TTCC, TTCC, TTCC, GGAA, TTCC, TTCC, TTCC, TTCC, TTCC, GGAA, GGAA, GGAA, TTCC, GGAA, TTCC, TTCC, GGAA, GGAA	136–139, 204–207, 420–423, 483–486, 528–531, 535–538, 720–723, 754–757, 773–776, 857–860, 879–882, 905–908, 930–933, 978–981, 1002–1005, 1132–1135, 1150–1153, 1236–1239, 1247–1250, 1356–1359
	GATA‐3	1	TTTTATCT	303–310
	cMyc	2	CACTTGA, CCAGGTG	759–765, 1138–1144
	NF‐kB	1	GGAAAAAGCCC	930–940
MST1	STAT‐5A	15	GGAA, GGAA, TTCC, GGAA, TTCC, GGAA, TTCC, TTCC, TTCC, GGAA, GGAA, GGAA, GGAA, TTCC, TTCC	188–191, 267–270, 394–397, 404–407, 512–515, 593–596, 798–801, 1057–1060, 1094–1097, 1167–1170, 1189–1192, 1205–1208, 1234–1237, 1274–1277, 1349–1352
	GATA‐3	2	TGTTATCT, GCCTATCT	108–115, 916–923
	cMyc	4	TCAGGTG, CCAGGTG, CACCTGC, CACCTGT	306–312, 321–327, 850–856, 1006–1012
	NF‐kB	1	AGGGAATTTTC	1165–1175
STK3	STAT‐5A	15	GGAA, TTCC, GGAA, TTCC, TTCC, GGAA, GGAA, GGAA, GGAA, GGAA, GGAA, GGAA, TTCC, TTCC, TTCC	81–84, 105–108, 175–178, 528–531, 639–642, 908–911, 1091–1094, 1126–1129, 1175–1178, 1190–1193, 1247–1250, 1269–1272, 1337–1340, 1354–1357, 1485–1488
	GATA‐3	2	AGTTATCT, ACCTATCT	785–792, 970–977
	cMyc	1	ACAAGTG	368–374
	NF‐kB	1	TGGGATTTTCC	632–642
SOS1	STAT‐5A	16	TTCC, GGAA, TTCC, TTCC, TTCC, GGAA, GGAA, GGAA, TTCC, TTCC, TTCC, GGAA, GGAA, TTCC, TTCC, TTCC	103–106, 247–250, 278–281, 321–324, 445–448, 564–567, 577–580, 704–707, 732–735, 921–924, 937–940, 962–965, 1033–1036, 1094–1097, 1118–1121, 1420–1423
	GATA‐3	4	AGATAATG, CGTTATCT, ACCTATCT, CCTTATCT	259–266, 332–339, 637–644, 687–694
	cMyc	1	CACCTGC	226–232
	NF‐kB	4	GGAAACACCGC, TGGCGCTTTCC, GGAAAACCCGG, CGGGCCTTTCC	577–587, 930–940, 962–972, 1111–1121
GAB1	STAT‐5A	20	GGAA, TTCC, TTCC, TTCC, GGAA, TTCC, TTCC, GGAA, GGAA, TTCC, TTCC, TTCC, TTCC, TTCC, GGAA, TTCC, GGAA, GGAA, GGAA, GGAA	211–214, 241–244, 258–261, 391–394, 428–431, 481–484, 524–527, 566–569, 730–733, 910–913, 996–999, 1016–1019, 1055–1058, 1153–1156, 1168–1171, 1214–1217, 1391–1394, 1453–1456, 1465–1468, 1474–1477
	GATA‐3	5	TAGTATCT, AACTATCT, TTTTATCT, AGATATTT, AGATAGAT	226–233, 314–321, 500–507, 571–578, 775–782
	cMyc	2	CACTTGT, GCAAGTG	65–71, 724–730
	NF‐kB	—	—	—
ETS1	STAT‐5A	19	TTCC, TTCC, TTCC, GGAA, TTCC, TTCC, GGAA, TTCC, TTCC, TTCC, TTCC, GGAA, TTCC, TTCC, TTCC, GGAA, TTCC, GGAA	260–263, 287–290, 341–344, 480–483, 531–534, 582–585, 597–600, 768–771, 796–799, 899–902, 927–930, 947–950, 954–957, 998–1001, 1277–1280, 1338–1341, 1396–1399, 1411–1414, 1466–1469
	GATA‐3	7	AGATATTT, CACTATCT, AGATAATG, AGATAAAA, AGATAAAG, AGATAAGC, AGTTATCT	317–324, 344–351, 464–471, 608–615, 985–992, 1005–1012, 1308–1315
	cMyc	2	GCATGTG, CACTTGA	216–222, 226–232
	NF‐kB	—	—	—

## 3 Discussion

This study gives the first integrated genomic and molecular overview of MAPK/ERK pathway genes in *B. bubalis*, linking structural, evolutionary and regulatory features with mammary biology. By combining phylogeny, synteny, motif and domain mapping, duplication and selection analysis and Transcription Factor Binding Site (TFBS) predictions, it outlines how ERK signalling may support mammary epithelial cell proliferation, differentiation, survival and milk protein transcription during lactation. Such roles have already been noted in mammary systems (Braicu et al. 2019), and more recent studies also point toward ERK/RSK signalling in oestrogen‐mediated regulation of mammary function (Wright and Lannigan 2023).

Phylogenetic relationships and synteny showed that buffalo MAPK/ERK genes are most closely aligned with *Bos taurus*, though lineage‐specific differences were also clear. In cattle, variation in ERK dynamics has been linked with shifts in downstream transcriptional programs (Wright and Lannigan 2023). The retention of synteny between buffalo and cattle points to a shared regulatory framework (Saleeb et al. 2019). Still, the distinct milk composition and yield seen in buffalo suggest additional fine‐tuning (Costa et al. 2020). Recent comparative studies further support this divergence (M. Zhou et al. 2021).

Motif and domain analysis confirmed that core kinase, ETS and Ras modules remain intact, while the absence of specific motifs in GRB2 and DUSP6 pointed to possible rewiring of signal transduction or feedback regulation. In mammary and epithelial models, reduced DUSP6 activity has been shown to prolong ERK phosphorylation (Hiratsuka et al. 2020). In contrast, elevated DUSP6 levels suppress pathway output and lower downstream transcriptional activity (Momeny et al. 2024). These motif differences in buffalo may therefore support a more sustained ERK signalling state, which could be favourable for milk production.

Ka/Ks values above 1 were detected for the JUN–ETS1 and DUSP6–MST1 (STK4) gene pairs, pointing toward adaptive evolution. JUN and ETS1 transcription factors work with hormone and growth factor signals to regulate casein and secretory gene networks (Pegolo et al. 2018). These regulators have also been linked with milk protein traits in cattle (A. Wang et al. 2024). MST1/2, as part of the Hippo pathway, interacts with ERK and YAP/TAZ to control differentiation and proliferation in glandular tissues (D. Wang et al. 2020). Such convergence has been highlighted in recent updates on mammary biology (Wright and Lannigan 2023).

Many ERK pathway proteins carried predicted disordered regions, hydrophilic profiles and high instability indices, features that support rapid partner exchange and turnover. Such flexibility is useful in mammary epithelial cells, where close coupling between hormonal or nutrient signals and transcription/translation quickly shapes milk output. ERK activity is also sensitive to redox balance in bovine systems (Zhang et al. 2017). Nutrient stress has been reported to influence ERK‐driven regulation of casein synthesis (M. Zhou et al. 2021), while hormonal signals further modulate mammary epithelial integrity through this pathway (Y. Li et al. 2024).

TFBS analysis showed dense STAT5A binding predictions across MAPK/ERK genes, consistent with bovine data where STAT5A directly drives casein expression (A. Wang et al. 2024). STAT5A is already established as a central regulator of lactogenic signalling (S.‐N. Lu et al. 2024). Enrichment of GATA‐3 motifs pointed toward developmental cross‐talk (Xue et al. 2023). c‐Myc motifs indicated links with growth and metabolic regulation, while NF‐κB motifs reflected connections with inflammatory signalling (Ivanova et al. 2021). Together, these patterns suggest that ERK signalling helps balance STAT5A‐driven milk synthesis with parallel stress and immune responses.

Heat stress in dairy ruminants disrupts mammary homeostasis, lowering milk yield and altering gene networks that include MAPK/ERK‐linked inflammatory and redox modules. Candidate nodes such as DUSP6 and JUN/ETS may influence buffalo resilience or susceptibility under these conditions (Lengi et al. 2022). Remodelling of MAPK/ERK‐related networks during heat stress has already been reported in bovine models (Zeng et al. 2023). Comparative work also shows that mammary adaptation to heat varies among ruminants, highlighting the importance of this pathway (Giannone et al. 2023, Xiong et al. 2025). In cattle, GWAS and expression studies have tied MAPK‐related regulators to milk traits (Pegolo et al. 2018), while in goats and sheep, transcriptome and noncoding RNA studies point to conserved regulatory plasticity during lactation (Marei et al. 2025).

The overlap of positive selection, motif variation and enriched TFBSs pointed to AP‐1/ETS, STAT5A and DUSP6–MST1/Hippo nodes as strong candidates for marker‐assisted selection or genomic prediction in buffalo. In cattle, the integration of mammary transcriptomics into breeding pipelines has already shown success (Pegolo et al. 2018). Similar pathway‐aware prediction strategies are now being emphasized for buffalo improvement programs (Wright and Lannigan 2023).

To confirm these computational predictions, future work should profile the expression of JUN, ETS1, DUSP6, MST1/2 and other MAPK targets in buffalo mammary epithelial cells across different lactation stages. Perturbation experiments using siRNA, CRISPRi, or pharmacological inhibition of DUSP6 would help test how feedback modulation influences casein transcription (Kobayashi et al. 2022). Chromatin‐level assays such as ChIP‐seq or ATAC‐seq could then map STAT5A and ETS/AP‐1 binding at buffalo milk‐protein and MAPK loci (A. Wang et al. 2024, Liu et al. 2025). Finally, buffalo or MEC models should be exposed to heat, oxidative stress or inflammatory challenges to see whether pathway variation predicts milk output or resilience against mastitis (Perez‐Hernandez et al. 2024).

In summary, embedding buffalo findings within bovine and broader ruminant frameworks highlights a conserved ERK–STAT5A axis for milk synthesis, while pointing to buffalo‐tuned regulators in AP‐1/ETS and DUSP6–MST1 feedback. These hypotheses are now testable and ready for translation into breeding and dairy management strategies. Integrating these candidate loci and regulatory elements into genomic prediction pipelines, mirroring the use of MAPK‐linked markers in cattle (Pegolo et al. 2018), could shift buffalo programs beyond basic production traits toward pathway‐aware selection, improving yield, composition and resilience under stress.

By combining whole‐genome resequencing, GWAS/QTL mapping and mammary transcriptomics, causal variants within MAPK/ERK genes and their regulatory regions can be identified. These variants can then be used in marker‐assisted selection, genomic estimated breeding values and haplotype‐based prediction models tailored to buffalo populations. Functional validation in mammary epithelial cells will be important to confirm functional alleles rather than just associated markers, improving the reliability of breeding choices.

Over time, such integrative approaches could support precision buffalo dairying, where genomic selection is paired with tailored nutrition, reproductive management and health strategies to enhance productivity and welfare under diverse farming systems. Comparative work with cattle, goats and sheep will also help benchmark beneficial regulatory variants in buffalo against conserved ruminant pathways, reducing risk and speeding adoption. Altogether, these findings place MAPK/ERK insights not only as a window into buffalo lactation biology but also as a practical framework for genomic selection in this species.

## 4 Materials and Methods

### 4.1 Genome Data Retrieval and Gene Identification

The genome sequences of *B. bubalis* (NDDB_SH_1), along with those of *B. taurus* (ARS‐UCD2.0), *Camelus bactrianus* (ASM4877302v1), *Odocoileus virginianus* (Ovbor_1.2), *Capra hircus* (ARS1.2) and *Homo sapiens* (GRCh38.p14), were retrieved from the NCBI Genome database (https://www.ncbi.nlm.nih.gov/). Human genome sequences were used as reference queries to extract genomic, proteomic and coding sequence data specific to the MAPK/ERK pathway genes in *B. bubalis*. The corresponding identification numbers for these genes are listed in Table 6. BLAST and Hidden Markov Model (HMM) tools were employed to retrieve the data, using an *E* value threshold of ≤ 1.0 × 10^−5^ (Altschul et al. 1990). Gene domains were annotated using the InterPro online platform (https://www.ebi.ac.uk/interpro/) (Eddy 2011). Duplicate sequences were removed to ensure a non‐redundant dataset.

**TABLE 6:** Data source, protein accession IDs, transcript IDs, gene IDs and genome IDs for the MAPK/ERK pathway genes in *B. bubalis*.

Gene name	Protein ID	Transcript ID	Gene ID	Genome ID	Data source
BRAF	XP_006078854.2	XM_006078792.3	102401079	NC_059164.1	NCBI
ARAF	XP_006058625.1	XM_006058563.4	102392863	NC_059181.1	NCBI
MAP2K1	XP_006050282.1	XM_006050220.4	102396842	NC_059167.1	NCBI
MAP2K2	XP_025149007.3	XM_025293222.3	102401744	NC_059165.1	NCBI
MAPK3	XP_045020224.1	XM_045164289.1	102403296	NC_059180.1	NCBI
MAPK1	XP_025123018.1	XM_025267233.2	102396206	NC_059173.1	NCBI
HRAS	XP_045022652.1	XM_045166717.1	112587129	NC_059165.1	NCBI
KRAS	XP_044797193.1	XM_044941258.2	102392995	NC_059160.1	NCBI
NRAS	XP_006064867.1	XM_006064805.4	102399487	NC_059162.1	NCBI
GRB2	XP_006045307.1	XM_006045245.3	102394713	NC_059159.1	NCBI
FOS	XP_006053957.1	XM_006053895.4	102393791	NC_059167.1	NCBI
JUN	XP_006048334.1	XM_006048272.4	102405224	NC_059162.1	NCBI
DUSP6	XP_006067080.1	XM_006067018.4	102391799	NC_059160.1	NCBI
ELK1	XP_044792750.1	XM_044936815.2	112577626	NC_059181.1	NCBI
ELK3	XP_044797416.1	XM_044941481.1	102397488	NC_059160.1	NCBI
ELK4	XP_025133521.1	XM_025277736.3	102403895	NC_059161.1	NCBI
MST1	XP_025128674.1	XM_025272889.1	102407730	NC_059177.1	NCBI
STK3	XP_025120861.1	XM_025265076.3	102414176	NC_059171.1	NCBI
SOS1	XP_006071264.1	XM_006071202.3	102392913	NC_059168.1	NCBI
GAB1	XP_006053863.1	XM_006053801.4	102390621	NC_059173.1	NCBI
ETS1	XP_006072436.3	XM_006072374.4	102389408	NC_059161.1	NCBI

To identify conserved domains in MAPK/ERK pathway genes, analyses were conducted using the Simple Modular Architecture Research Tool (SMART) (http://smart.embl‐heidelberg.de/) (Letunic et al. 2021) and the NCBI Conserved Domain Database (CDD) (https://www.ncbi.nlm.nih.gov/Structure/cdd/cdd.shtml) (S. Lu et al. 2020).

### 4.2 Physicochemical Characterization of Genes

The physicochemical properties of the identified proteins, including molecular weight (MW), pI, grand average of hydropathy (GRAVY), AI and II, were assessed using the Expasy ProtParam tool (https://web.expasy.org/protparam/) (Gasteiger et al. 2005). Chromosome numbers were determined by analysing genomic sequence data through the NCBI database.

### 4.3 Phylogenetic Analysis

The evolutionary relationships between *B. bubalis* and the reference species (*B. taurus*, *C. bactrianus*, *O. virginianus*, *C. hircus* and *H. sapiens*) were investigated using the ITOL platform (https://itol.embl.de/login.cgi) (Letunic and Bork 2021). Phylogenetic trees were constructed based on genetic and physical similarities, which allowed us to explore the evolutionary divergence and common ancestry of the MAPK/ERK pathway genes across the species.

### 4.4 Chromosomal Mapping and Gene Duplication

The duplication events of MAPK/ERK pathway genes in *B. bubalis* were analyzed through pairwise alignment of gene sequences using the MUSCLE algorithm implemented in MEGA11 v.11.0 (Tamura et al. 2021). The rates of synonymous substitutions per synonymous site (Ks) and non‐synonymous substitutions per non‐synonymous site (Ka) were calculated using TBtools (https://github.com/CJ‐Chen/TBtools) (Chen et al. 2020). Gene duplication age was estimated in million years ago (MYA) using the formula *t* = Ks/2*r*, where the mutation rate (*r*) is 1.1 × 10^−8^ (Lynch and Conery 2000).

### 4.5 Synteny Analysis

To investigate synteny, the MAPK/ERK pathway genes of *B. bubalis* and *H. sapiens* were analysed using the MCScanX algorithm implemented in TBtools, with a BLAST hit threshold of 5 and an *E* value cutoff of 1e^−10^. Chromosomal locations of the genes within the *B. bubalis* genome were determined using genomic data and General Feature Format (GFF) files with TBtools as the visualization tool. These chromosomal data were further used to create Circos plots, which provided a visual representation of genomic organization and positional relationships among the MAPK/ERK pathway genes in *B. bubalis* (Chen et al. 2020).

### 4.6 Investigation of Structural Properties

Conserved motifs in the MAPK/ERK pathway genes were identified using the MEME Suite (https://meme‐suite.org/meme/), which detected 10 distinct and conserved motif patterns (Bailey et al. 2015). For domain analysis, data obtained from the CDD (https://www.ncbi.nlm.nih.gov/Structure/cdd/wrpsb.cgi) were used in conjunction with TBtools (S. Lu et al. 2020). Gene structure visualization was performed in TBtools using the GTF file, enabling the representation of exon‐intron arrangements. TBtools was then utilized to integrate the results of the conserved motif analysis, domain identification and gene structure visualization. This integration, along with a phylogenetic tree, produced a comprehensive graphical representation, highlighting the conserved motifs, functional domains and exon‐intron structures of the MAPK/ERK pathway genes (Chen et al. 2020).

### 4.7 2D and 3D Models of Proteins

Protein structure analysis was conducted using the PSIPRED online tool (http://bioinf.cs.ucl.ac.uk/psipred/), recognized for its accuracy in predicting secondary structures (Jones 1999). Additionally, three‐dimensional protein models were generated with Phyre2 (https://www.sbg.bio.ic.ac.uk/~phyre2/html/page.cgi?id=index), a web‐based platform specializing in homology‐based protein modelling (Kelley et al. 2015). The resulting models were further validated using AlphaFold2 (https://alphafold.ebi.ac.uk/) to ensure structural reliability (Varadi et al. 2024).

### 4.8 Prediction of Intra‐Domains in Proteins

The Scan Prosite tool (https://prosite.expasy.org/scanprosite/) was used to detect functional and structural intra‐domains within the protein. The protein sequence was input into the platform to search and analyse motifs from the Prosite database (Sigrist et al. 2002).

### 4.9 TFBSs Identification

Promoter sequences of 1500 bp upstream of each gene were analysed using PROMO software (https://alggen.lsi.upc.es/cgi‐bin/promov3/promo/promoinit.cgi?dirDB=TF8.3), which employs version 6.4 of the TRANSFAC database to identify potential TFBSs (Farre 2003). The analysis focused on four key binding sites: GATA‐3, STAT‐5A, NF‐κB and c‐Myc. GATA‐3, known for its role in mammary gland development, can also be activated through the MAPK/ERK signalling pathway. It helps maintain cellular differentiation and functionality in lactating tissues, essential for continuous milk secretion (Yoon et al. 2010). NF‐κB is often activated during lactation, contributing to cell survival and immune responses in the mammary gland (Connelly et al. 2010). STAT‐5A, activated by prolactin, plays a vital role in lactation by driving the expression of genes involved in milk protein synthesis and ensuring the survival of mammary gland cells (Zaki et al. 2022). c‐Myc plays a crucial role in the proliferation and differentiation of mammary epithelial cells, which are essential for effective lactation (Stoelzle et al. 2009).

## 5 Conclusion

In summary, this study provides a comprehensive, genome‐wide analysis of the MAPK/ERK pathway genes in *B. bubalis*, highlighting their crucial roles in regulating milk production. By examining the diversity, structure and functions of these genes, we have gained valuable insights into their evolutionary history, shaped by genetic variation, gene duplication and selective forces. The integration of phylogenetic analysis with genome characterization emphasizes the importance of MAPK/ERK pathway genes in the regulation of milk production in *B. bubalis*. These findings not only enhance our understanding of the evolutionary significance of these genes but also offer important genetic resources for future efforts in genetic improvement and sustainable management of *B. bubalis* populations. This work paves the way for future studies focused on leveraging these genetic insights to improve milk yield and overall productivity in *B. bubalis*, supporting the development of more effective breeding strategies.

## Author Contributions

**Saima Naz**: conceptualization, investigation. **Urwah Ishaque**: methodology, validation. **Ahmad Manan Mustafa Chatha**: software, data curation. **Babar Maqbool**: writing – review and editing. **Qudrat Ullah**: visualization, validation, formal analysis. **Muhammad Farooq**: writing – original draft, investigation. **Shabana Naz**: writing – review and editing, writing – original draft. **Naseer Khan Momand**: software, data curation. **Ibrahim A. Alhidary**: resources.

## Funding

We extend our appreciation to the Ongoing Research Funding (ORF‐2025‐833), King Saud University, Riyadh, Saudi Arabia.

## Ethics Statement

The authors have nothing to report.

## Consent

The authors have nothing to report.

## Conflicts of Interest

The authors declare no conflicts of interest.

## Supporting information




**Table S1**: Genomic properties of MAPK/ERK pathway genes in B. bubalis, retrieved from the NCBI database.
**Figure S1**: PSIPRED‐predicted 2D structures of 21 genes associated with the MAPK/ERK pathway, illustrating their roles in regulating milk production in *B. bubalis*. The genes are labeled alphabetically as follows: a. BRAF, b. ARAF, c. MAP2K1, d. MAP2K2, e. MAPK3, f. MAPK1, g. HRAS, h. KRAS, i. NRAS, j. GRB2, k. FOS, l. JUN, m. DUSP6, n. ELK1, o. ELK3, p. ELK4, q. MST1, r. STK3, s. SOS1, t. GAB1, and u. ETS1.
**Figure S2**: AlphaFold2‐Based 3D Modeling of MAPK/ERK Pathway Genes in *B. bubalis*.

## Data Availability

The data that support the findings of this study are available from the corresponding author upon reasonable request.
