# Supplementary material for: Regulation of Milk Production by the MAPK/ERK Pathway in Water Buffalo (Bubalus bubalis): Genomic and Molecular Insights
Source: Vet Med Sci. 2025 Nov 20;11(6):e70703. doi: 10.1002/vms3.70703 (PMC12631544; doi:10.1002/vms3.70703)
Supplement: Supplementary file 1 — Table S1: Genomic properties of MAPK/ERK pathway genes in B. bubalis, retrieved from the NCBI database. Figure S1: PSIPRED‐predicted 2D structures of 21 genes associated with the MAPK/ERK pathway, illustrating their roles in regulating milk production in B. bubalis. The genes are labeled alphabetically as follows: a. BRAF, b. ARAF, c. MAP2K1, d. MAP2K2, e. MAPK3, f. MAPK1, g. HRAS, h. KRAS, i. NRAS, j. GRB2, k. FOS, l. JUN, m. DUSP6, n. ELK1, o. ELK3, p. ELK4, q. MST1, r. STK3, s. SOS1, t. GAB1, and u. ETS1. Figure S2: AlphaFold2‐Based 3D Modeling of MAPK/ERK Pathway Genes in B. bubalis. [file VMS3-11-e70703-s001.docx]

**Table S1.** Genomic properties of MAPK/ERK pathway genes in *B. bubalis*, retrieved from the NCBI database.

| **Gene Name** | **Gene Symbol** | **Gene Length (nt)** | **Chromosome Length (bp)** | **Gene Location on Chr. (start)** | **Gene Location on Chr. (End)** | **Strand Direction** |
| --- | --- | --- | --- | --- | --- | --- |
| Serine/Threonine Kinase B-Raf | BRAF | 9582 | 119318788 | 103475022 | 103640973 | R |
| Serine/Threonine Kinase A-Raf | ARAF | 2461 | 143192433 | 56741854 | 56752466 | R |
| Mitogen-Activated Protein Kinase Kinase 1 | MAP2K1 | 2533 | 102416932 | 89483834 | 89557338 | R |
| Mitogen-Activated Protein Kinase Kinase 2 | MAP2K2 | 1552 | 110262714 | 91286096 | 91309185 | R |
| Mitogen-Activated Protein Kinase 3 | MAPK3 | 1841 | 42172481 | 16100332 | 16107168 | R |
| Mitogen-Activated Protein Kinase 1 | MAPK1 | 4437 | 72603365 | 857777 | 914148 | R |
| GTPase HRas | HRAS | 695 | 110262714 | 95812458 | 95813381 | R |
| GTPase KRas | KRAS | 5389 | 164971372 | 35048964 | 35091755 | R |
| GTPase NRas | NRAS | 4263 | 120418900 | 28787337 | 28797249 | F |
| Growth Factor Receptor-Bound Protein 2 | GRB2 | 2870 | 174872328 | 7132402 | 7200000 | R |
| Proto-Oncogene Fos | FOS | 2126 | 102416932 | 16803081 | 16806547 | R |
| Proto-Oncogene Jun | JUN | 2715 | 120418900 | 86995188 | 86997902 | F |
| Dual Specificity Phosphatase 6 | DUSP6 | 2900 | 164971372 | 100968314 | 100972854 | F |
| ELK1, ETS Transcription Factor | ELK1 | 3826 | 143192433 | 56656551 | 56673186 | F |
| ELK3, ETS Transcription Factor | ELK3 | 3781 | 164971372 | 59595717 | 59772882 | R |
| ELK4, ETS Transcription Factor | ELK4 | 9272 | 132500020 | 77866405 | 77885020 | F |
| Macrophage Stimulating 1 | MST1 | 2303 | 60624307 | 50409934 | 50414872 | F |
| Serine/Threonine Kinase 3 | STK3 | 2976 | 81832314 | 16496776 | 16802073 | R |
| Son of Sevenless Homolog 1 | SOS1 | 5197 | 106386409 | 21056414 | 21187830 | R |
| GRB2-Associated Binding Protein 1 | GAB1 | 4966 | 72603365 | 57819649 | 57946995 | F |
| ETS Proto-Oncogene 1, Transcription Factor | ETS1 | 5173 | 132500020 | 112510450 | 112649188 | R |

**a.**


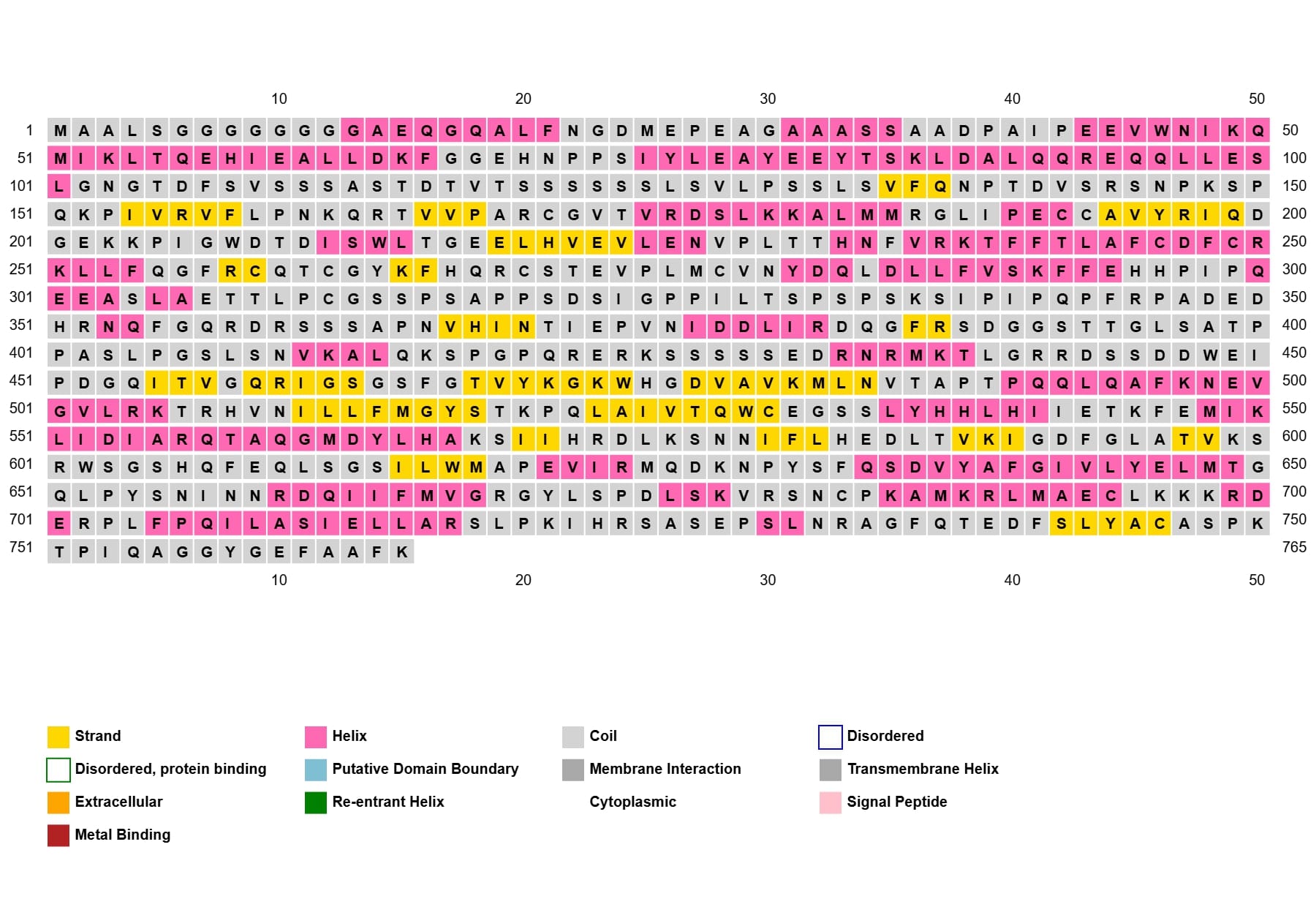


**b.**


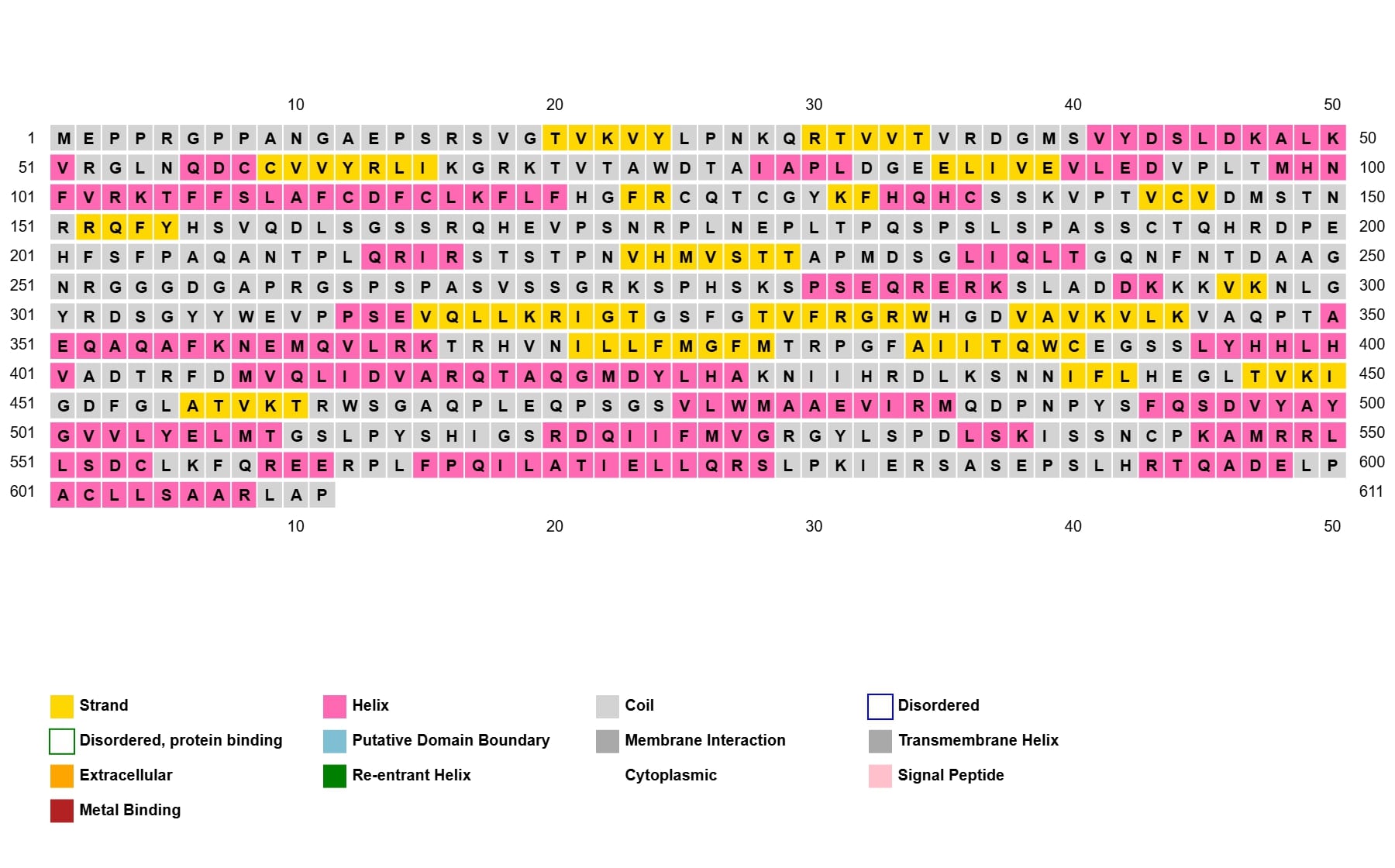


**c.**


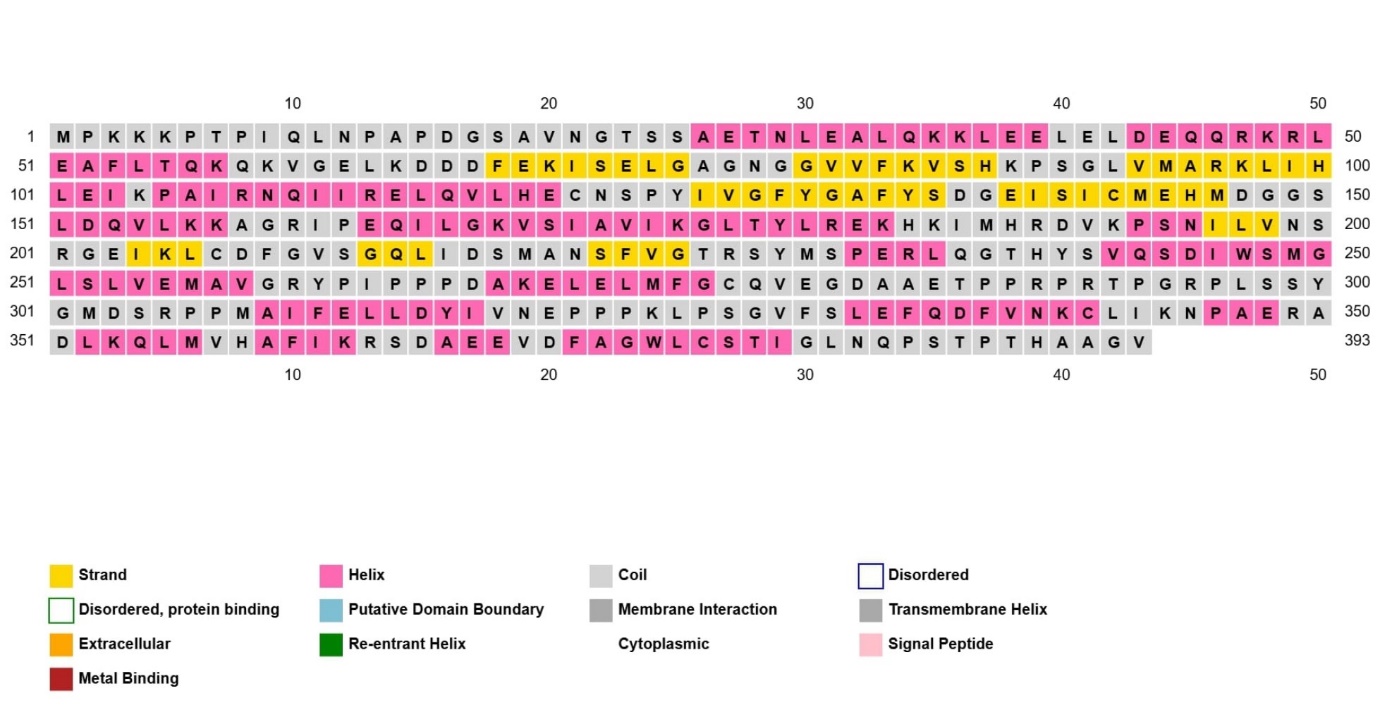


**d.**


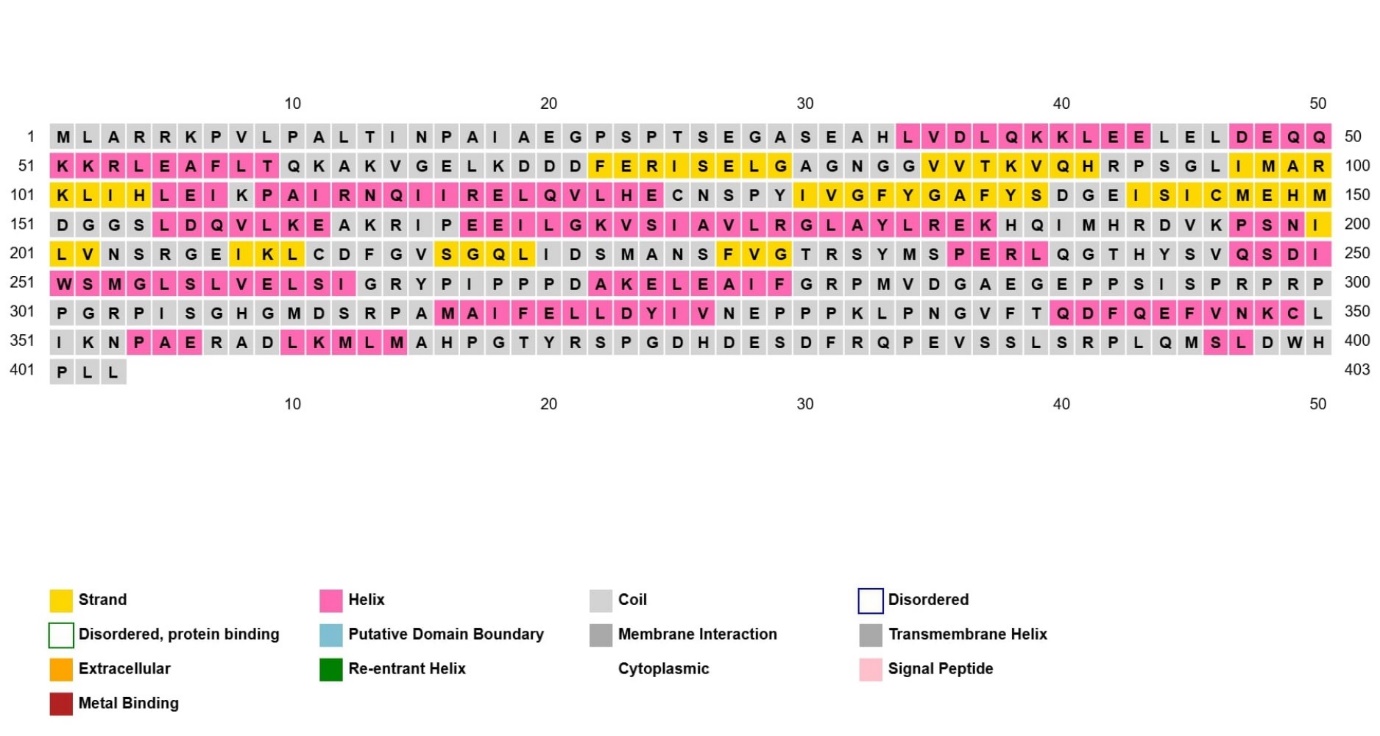


**e.**


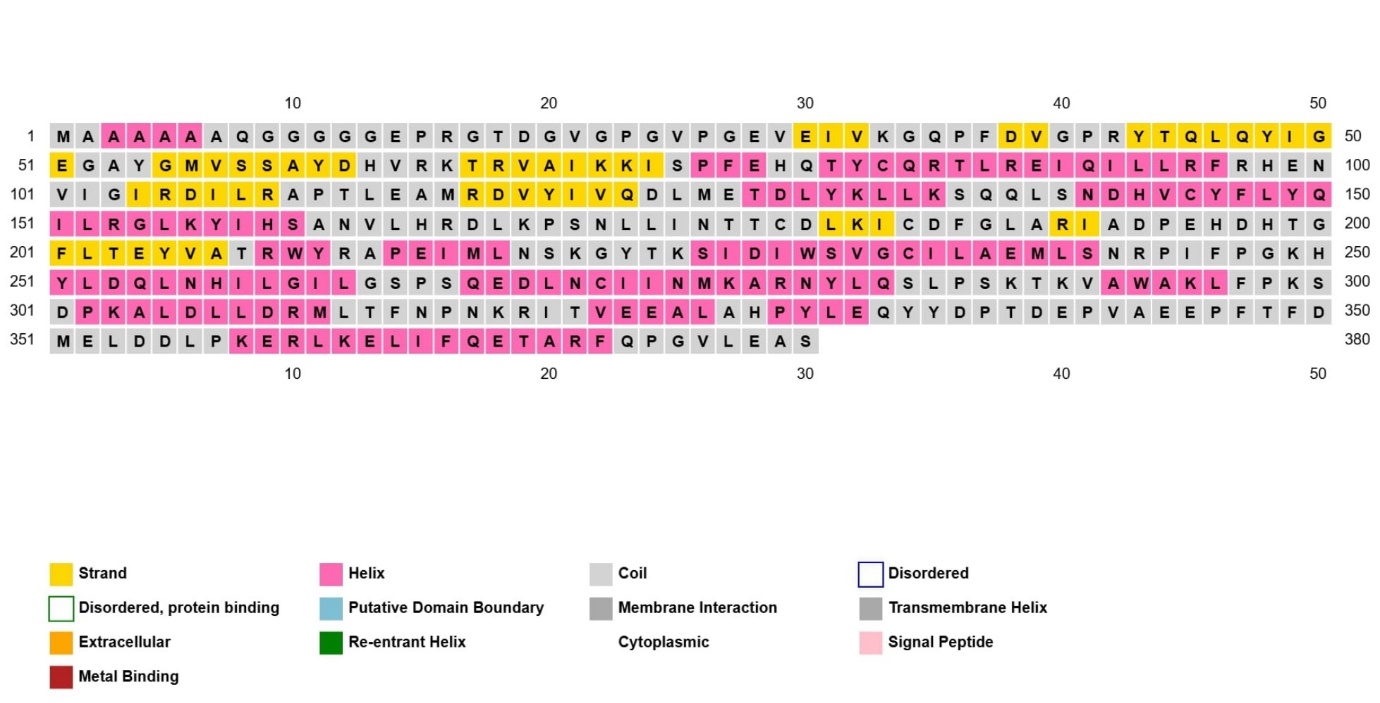


**f.**


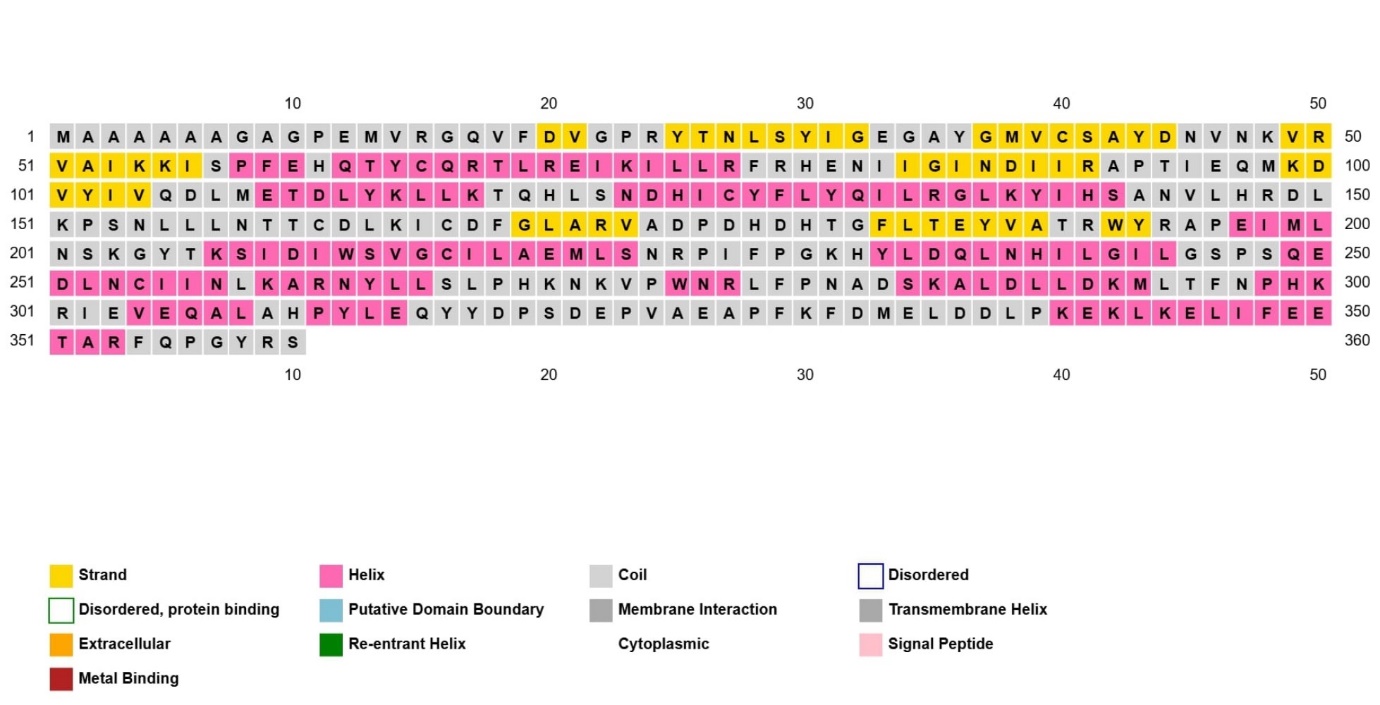


**g.**


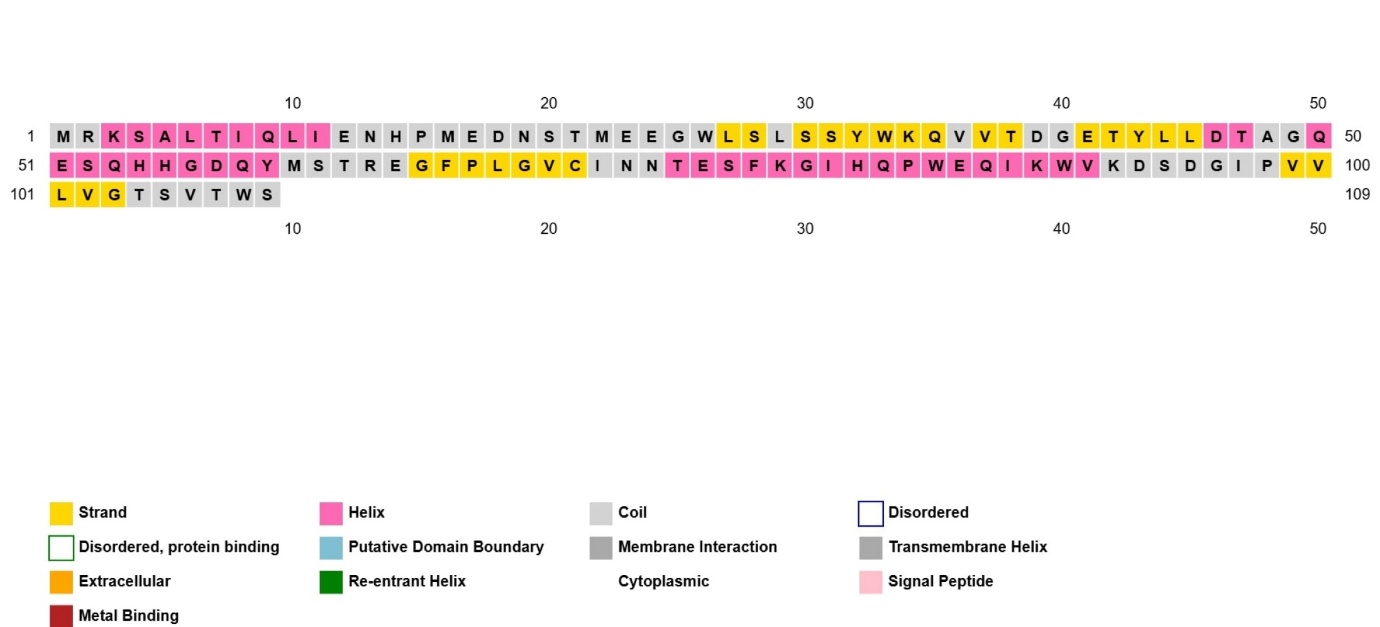


**h.**


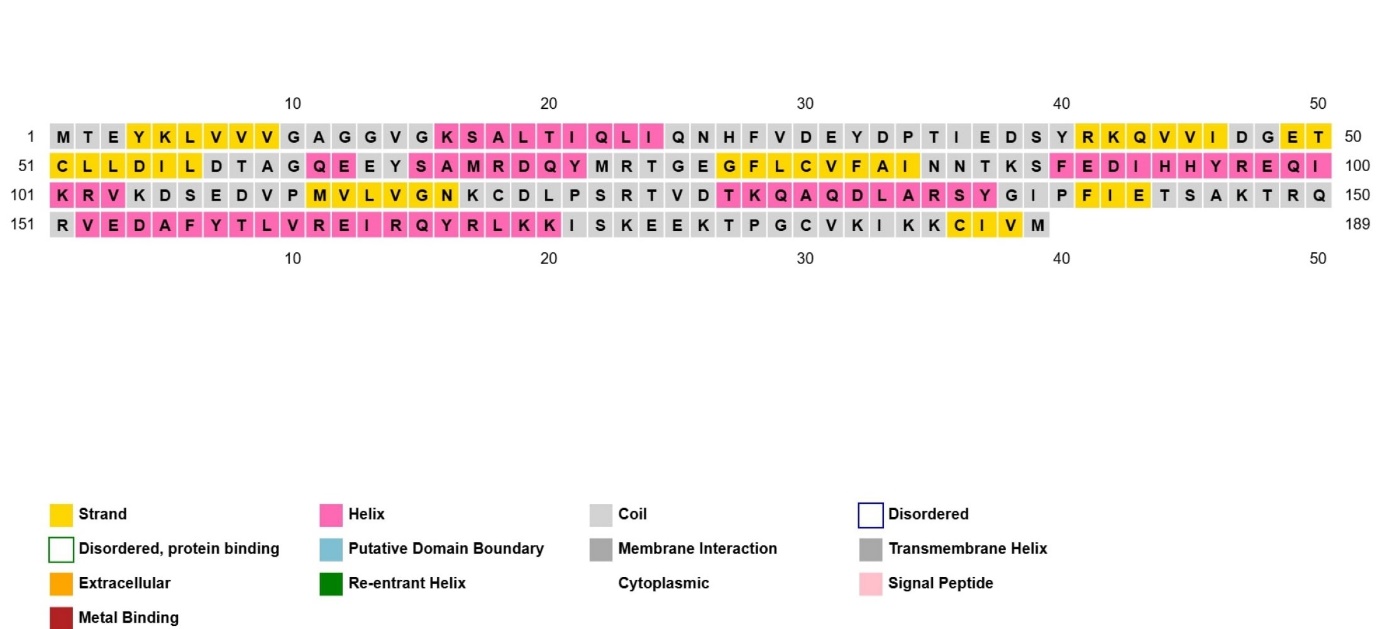


**i.**


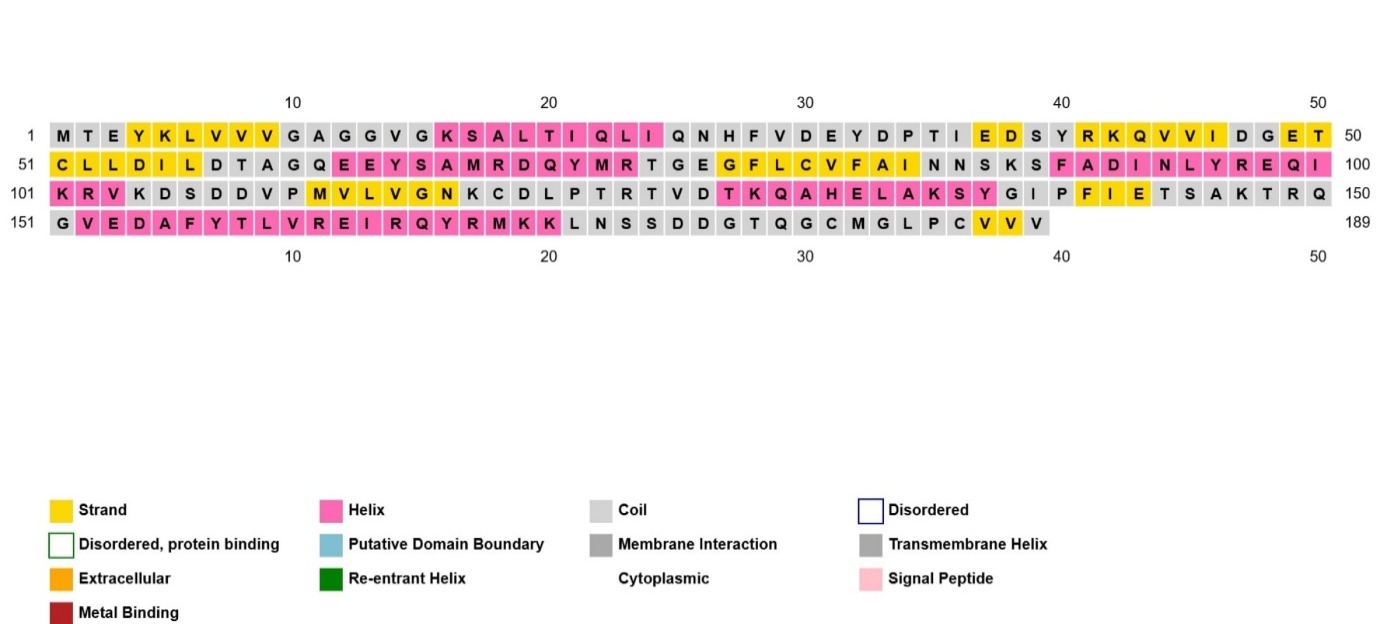


**j.**


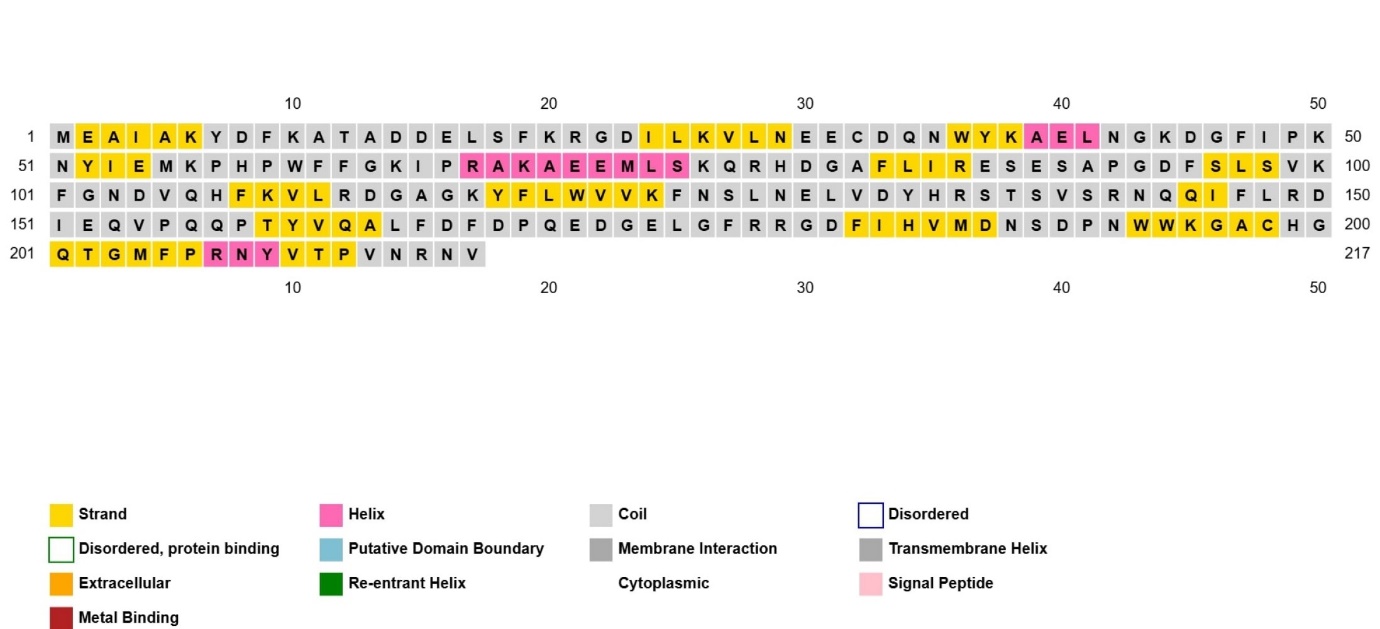


**k.**


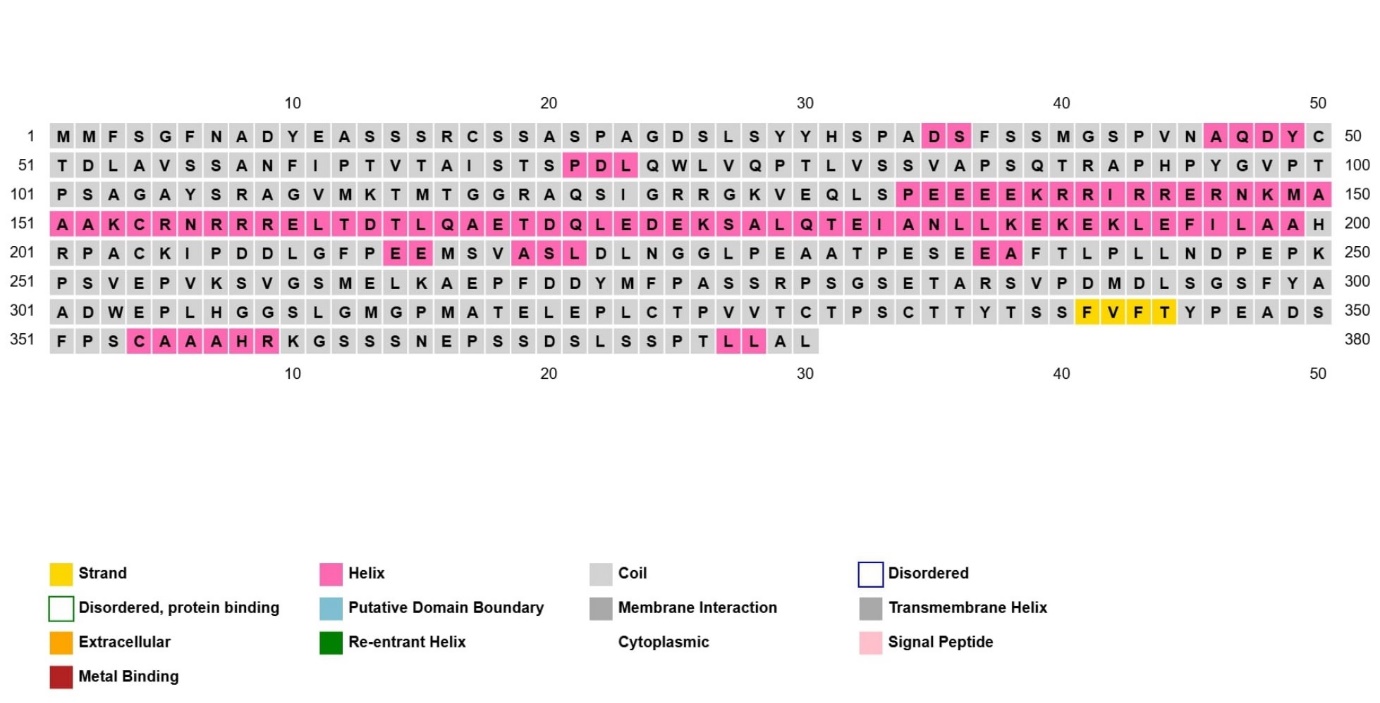


**l.**


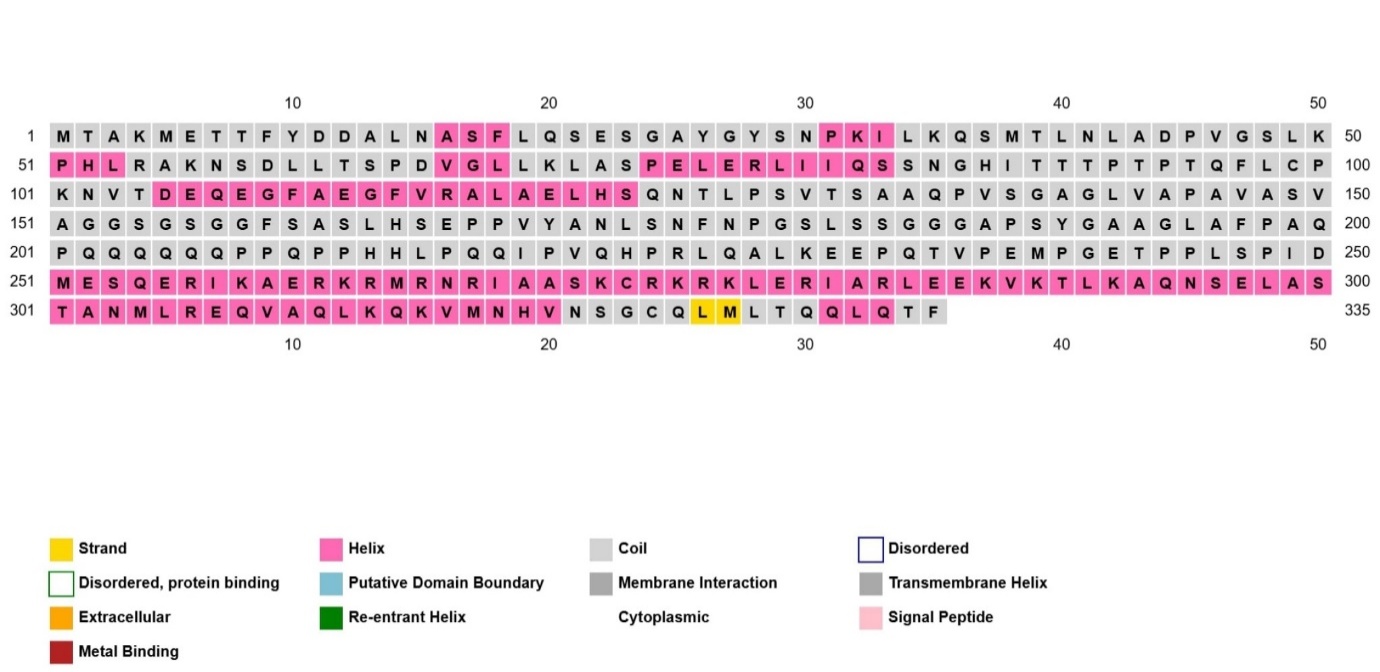


**m.**


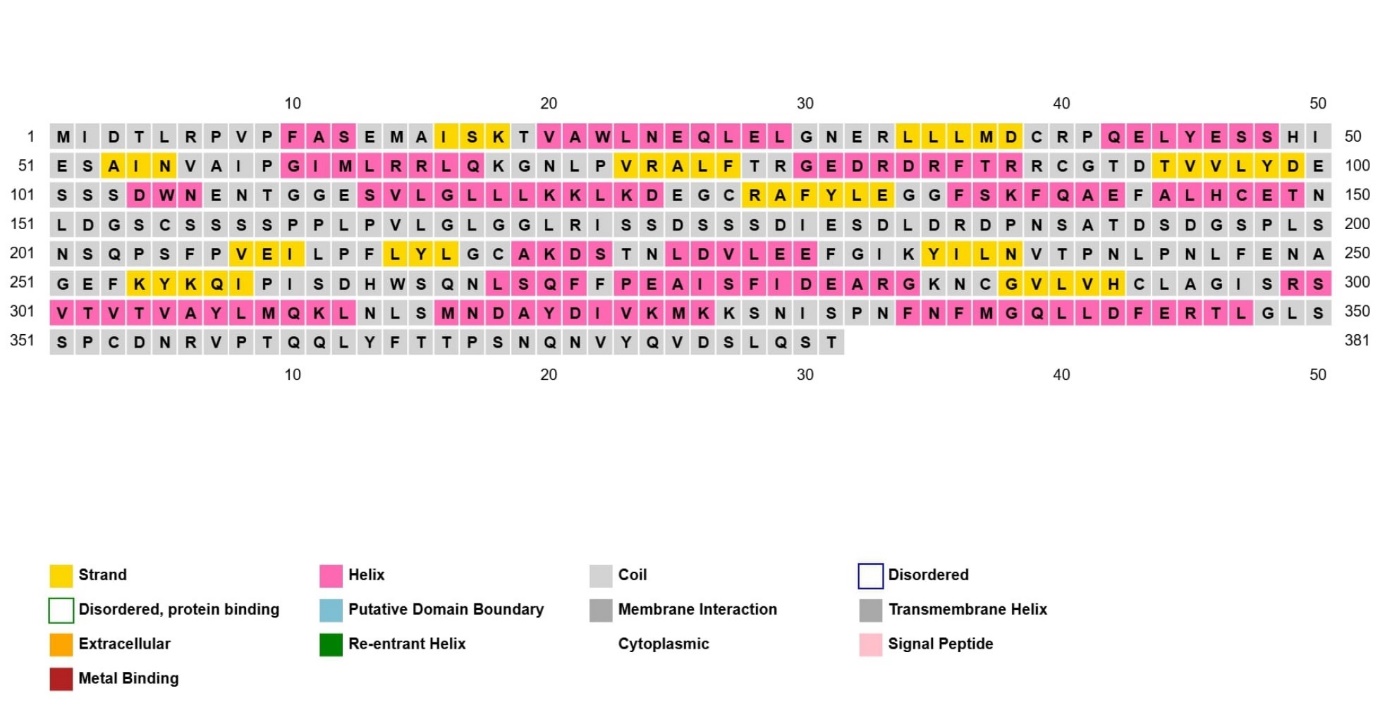


**n.**


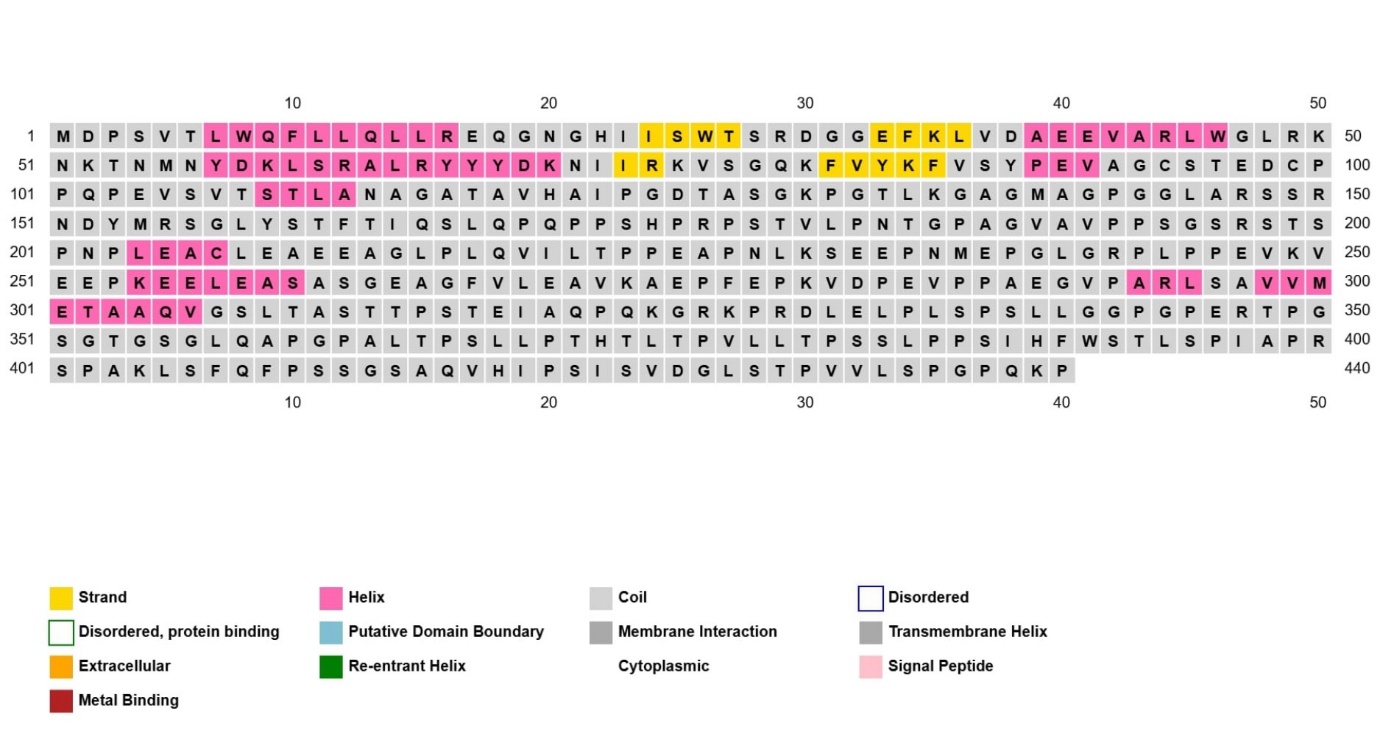


**o.**


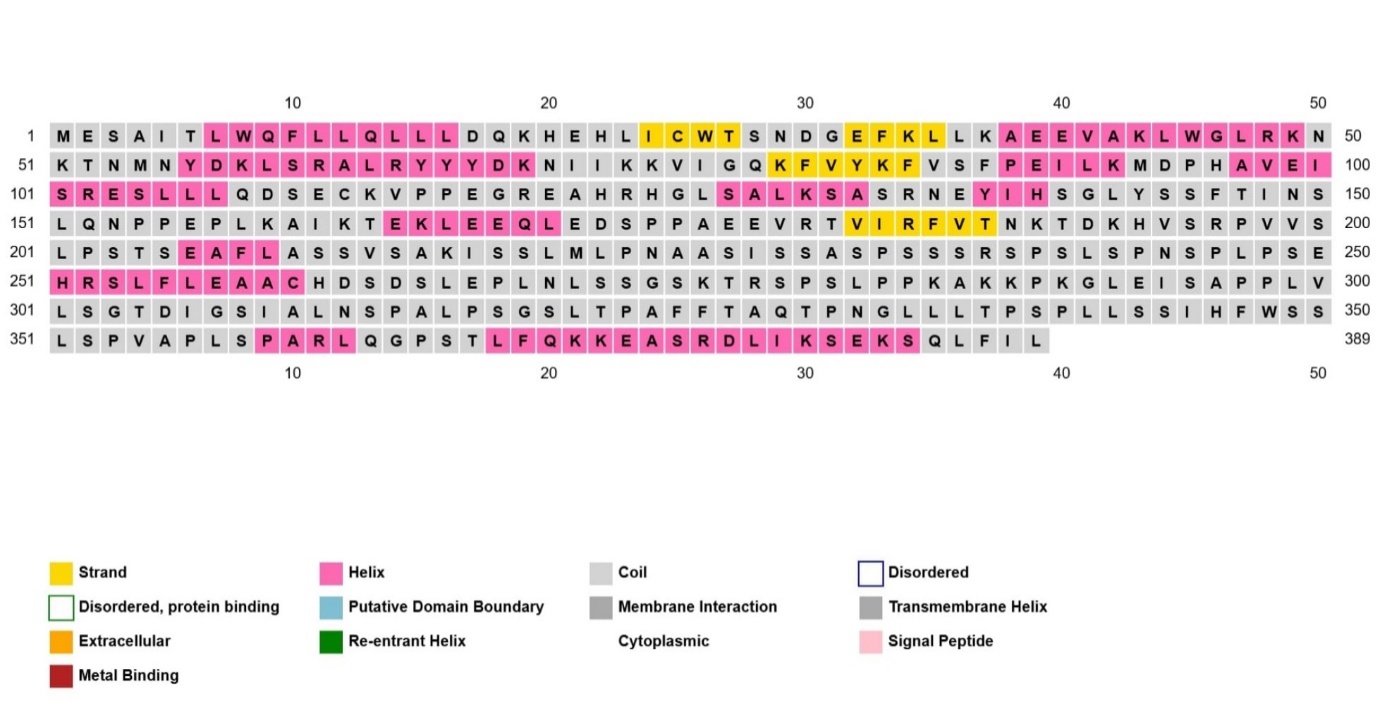


**p.**


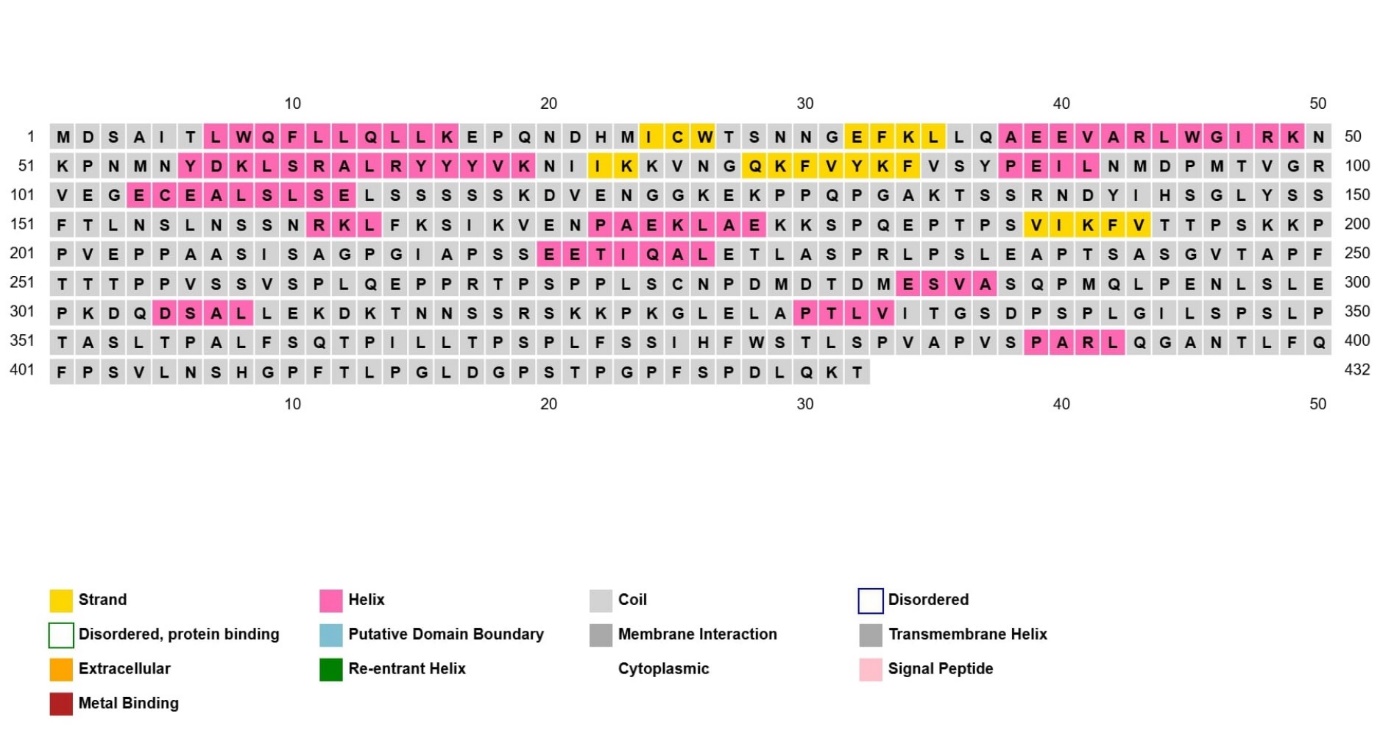


**q.**


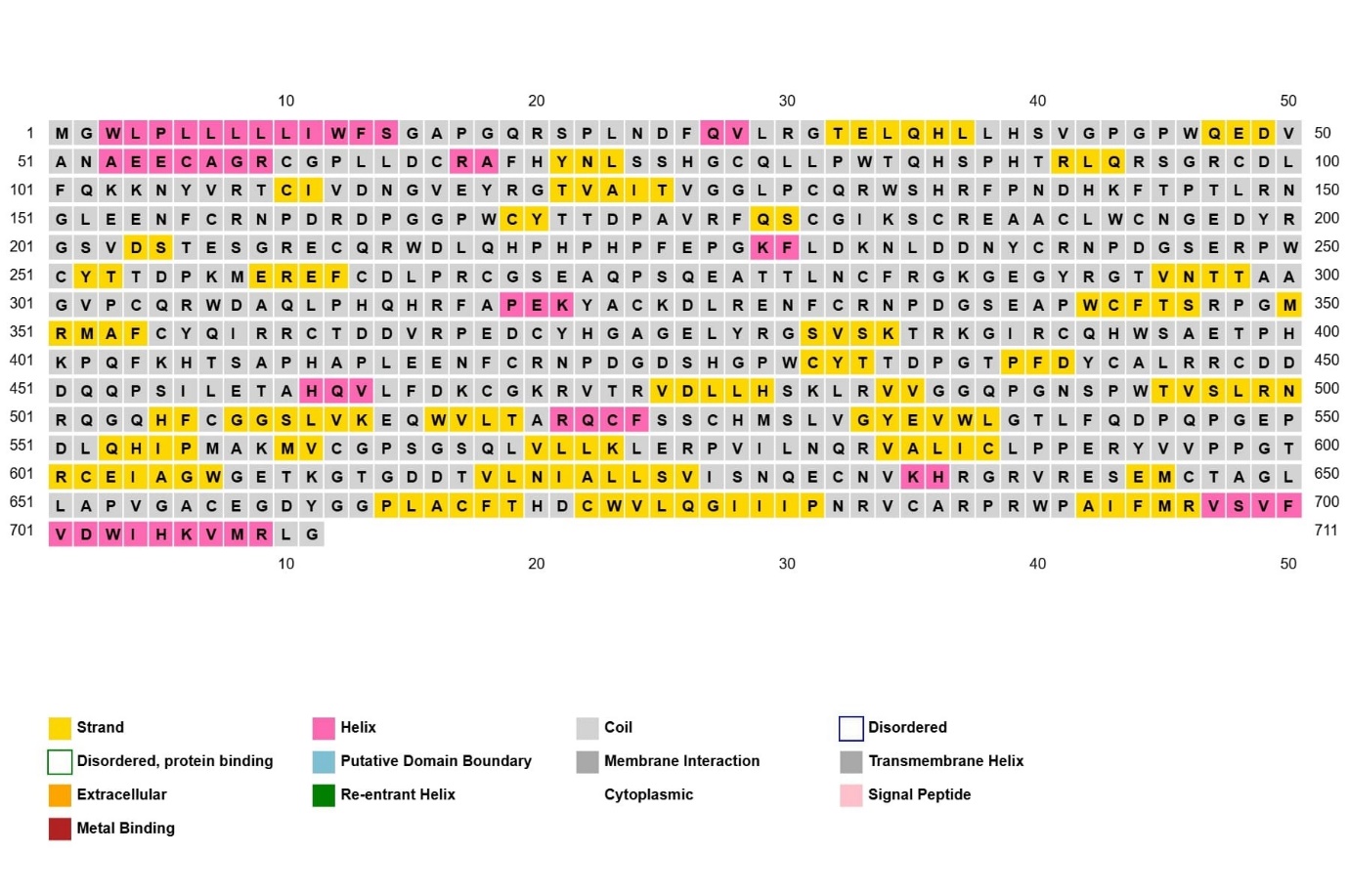


**r.**


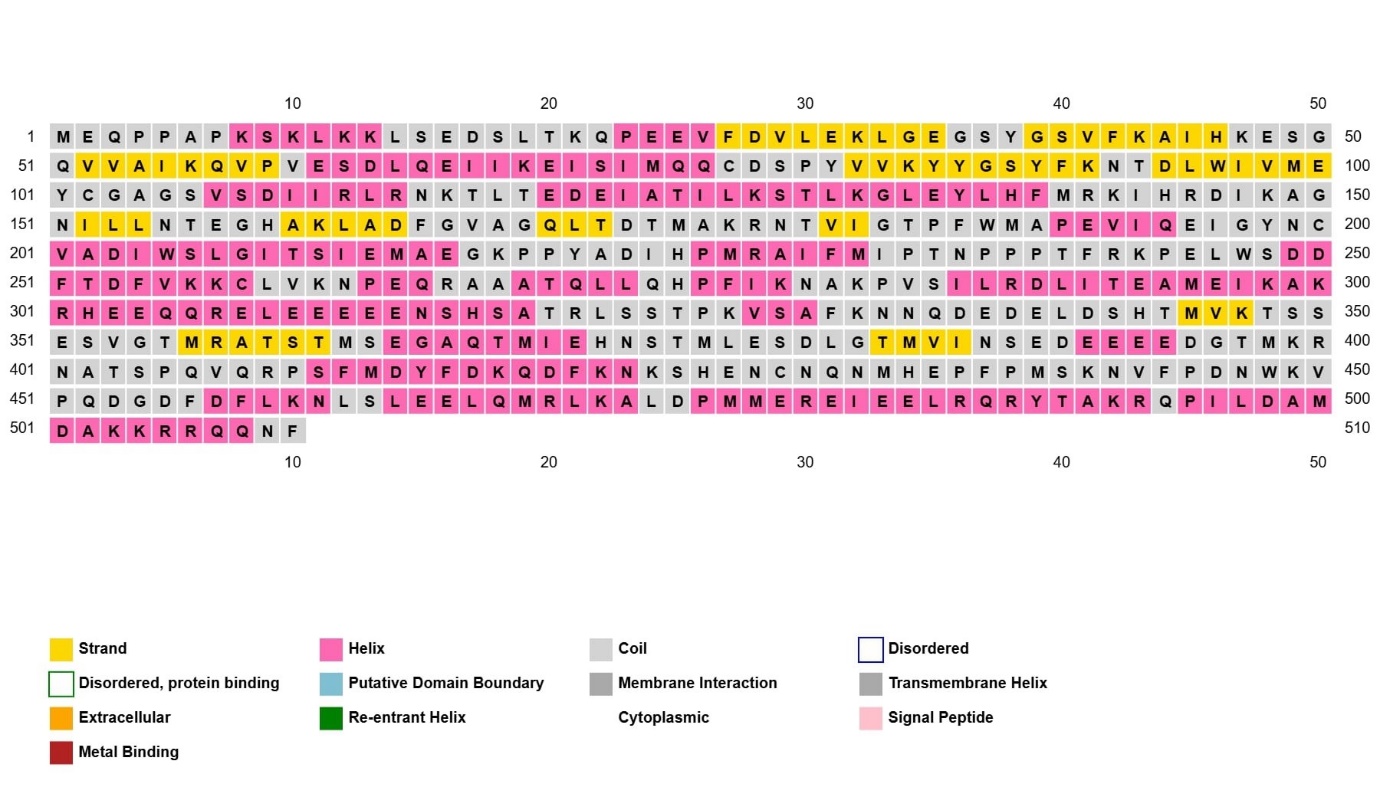


**s.**


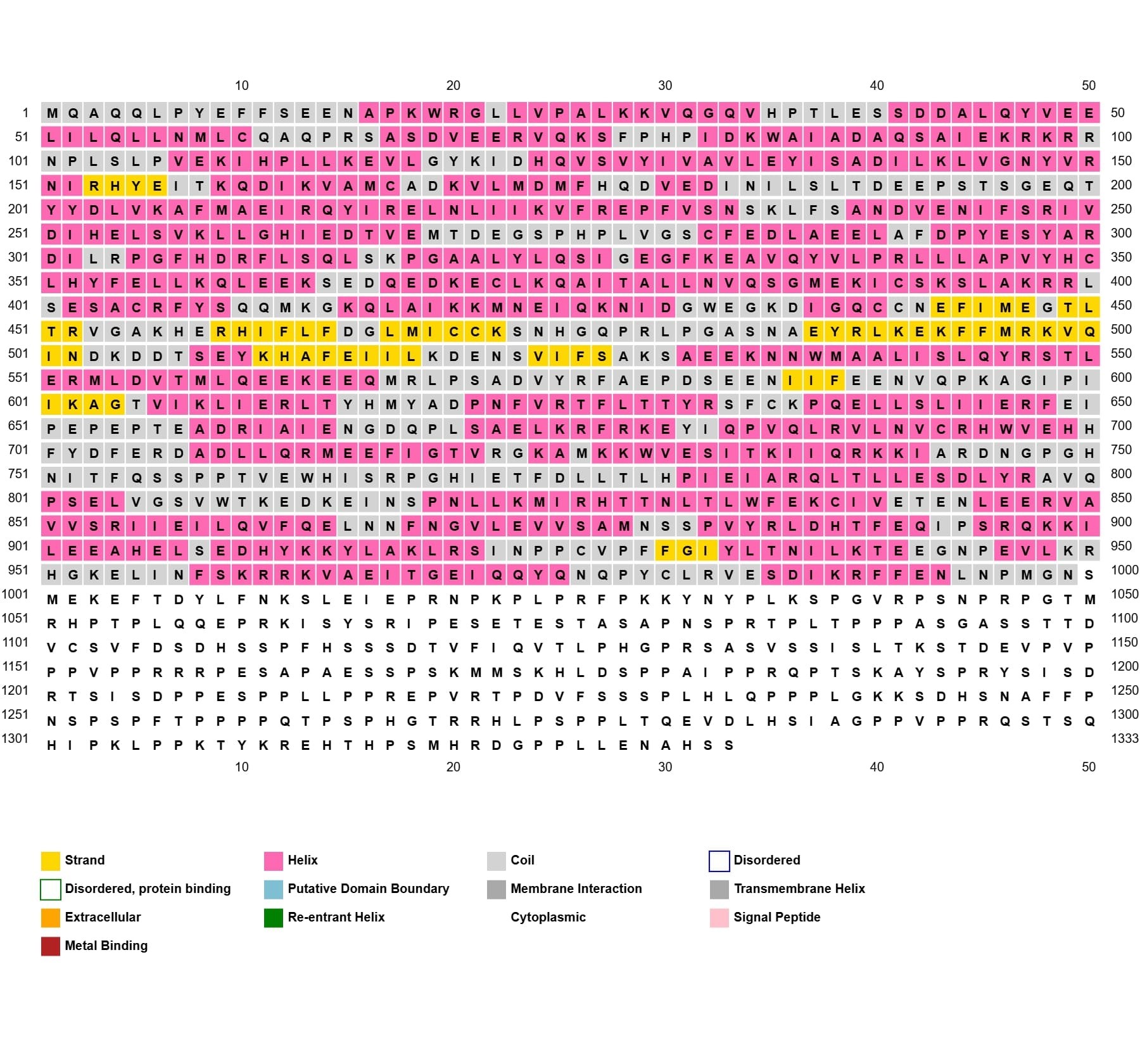


**t.**


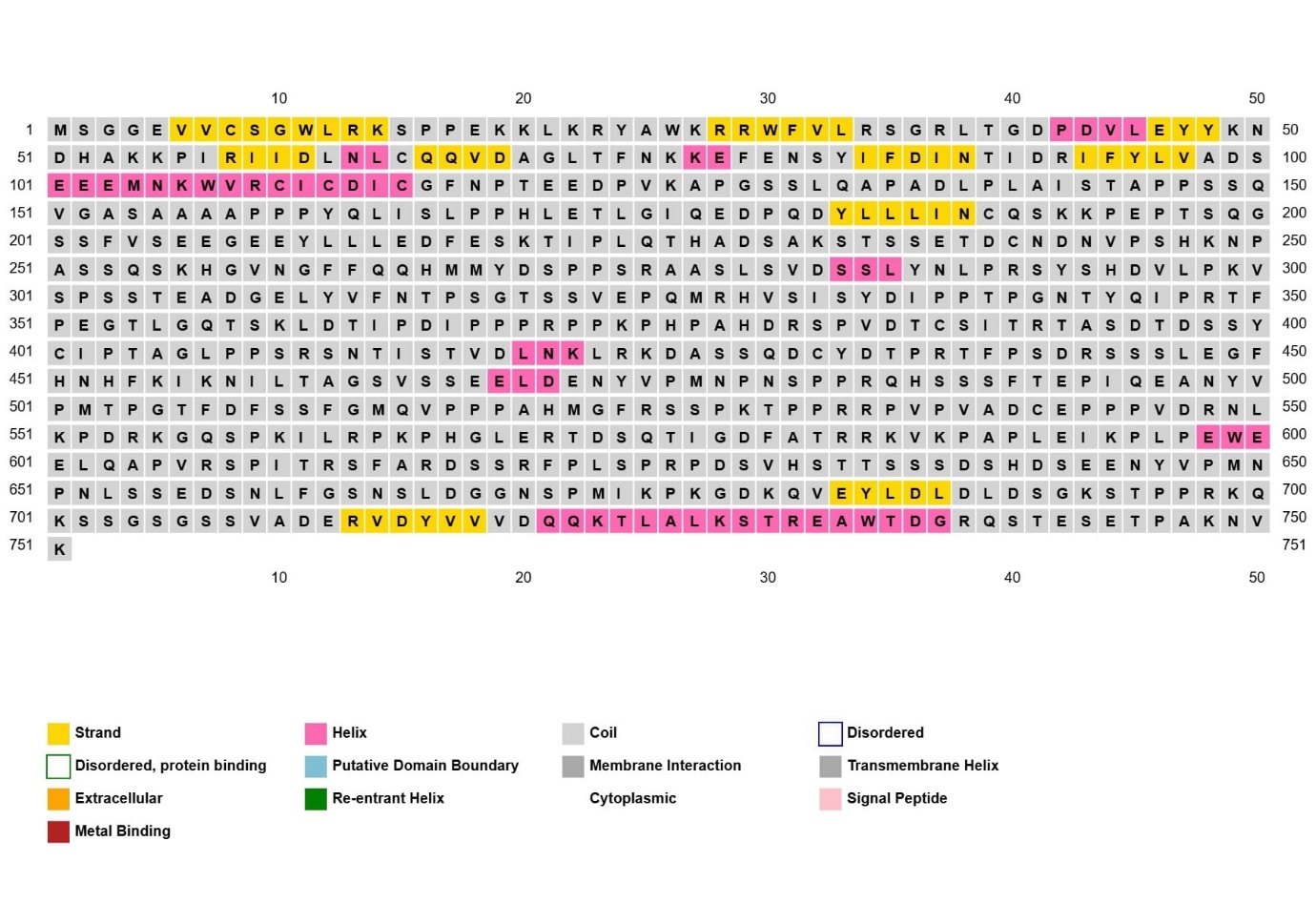


**u.**


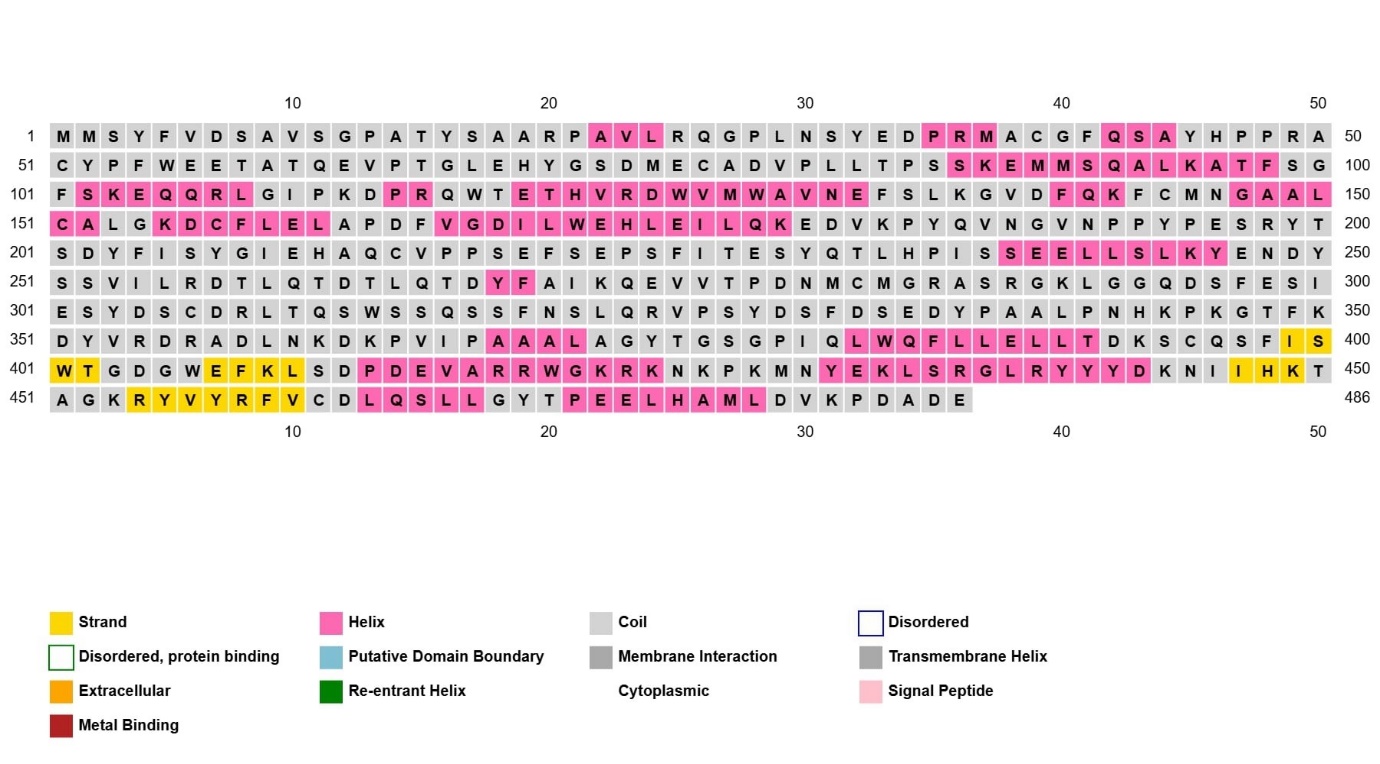


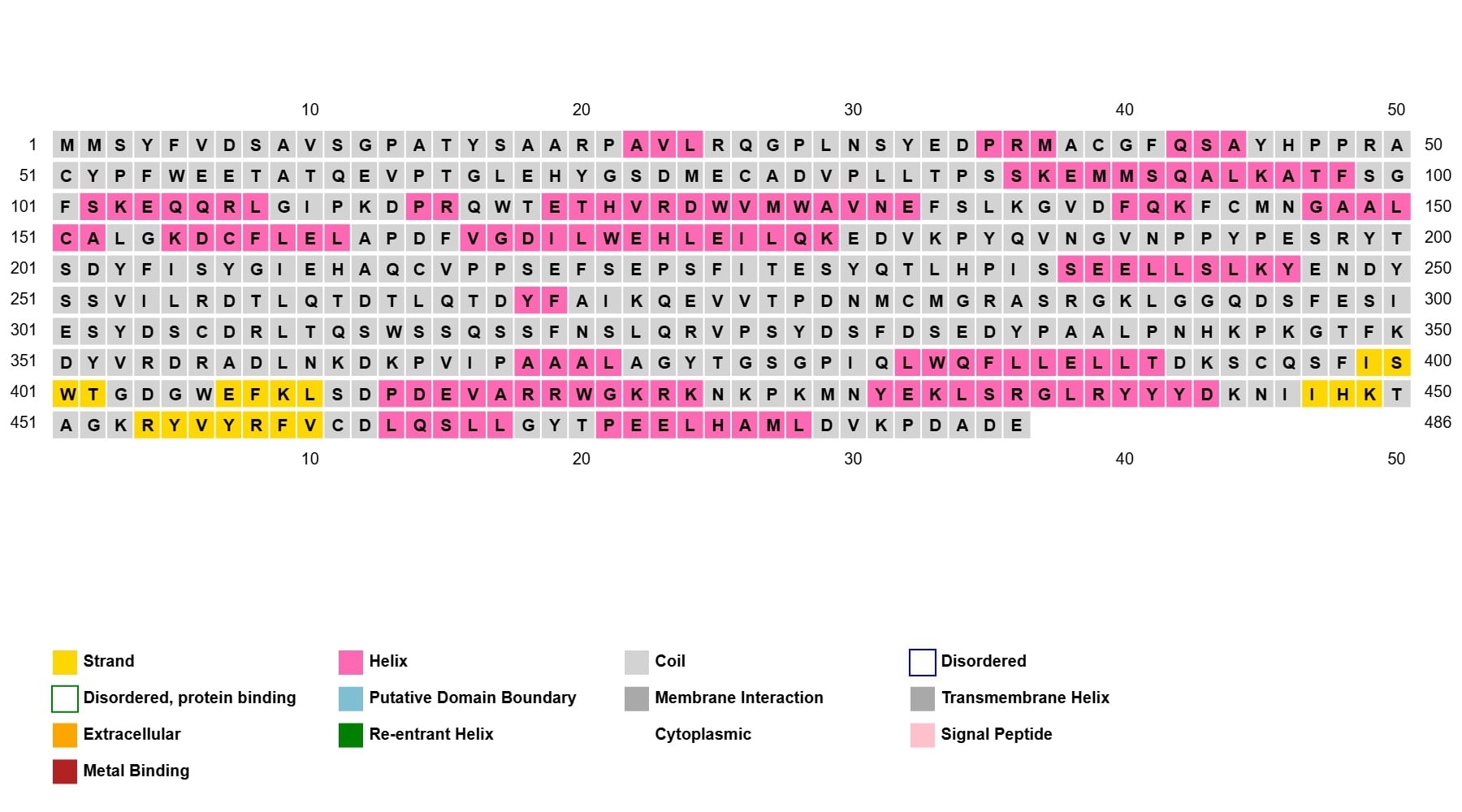


**Figure S1.** PSIPRED-predicted 2D structures of 21 genes associated with the MAPK/ERK pathway, illustrating their roles in regulating milk production in *B. bubalis*. The genes are labeled alphabetically as follows: a. BRAF, b. ARAF, c. MAP2K1, d. MAP2K2, e. MAPK3, f. MAPK1, g. HRAS, h. KRAS, i. NRAS, j. GRB2, k. FOS, l. JUN, m. DUSP6, n. ELK1, o. ELK3, p. ELK4, q. MST1, r. STK3, s. SOS1, t. GAB1, and u. ETS1.


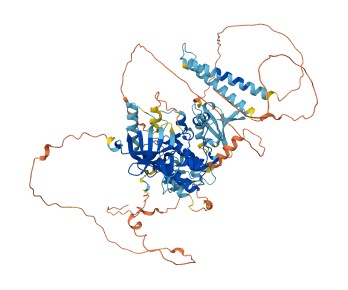

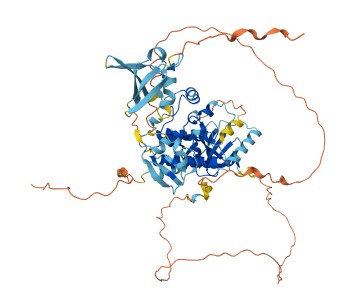

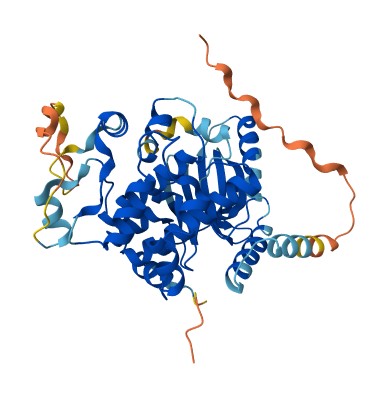

 **BRAF ARAF MAP2K1**


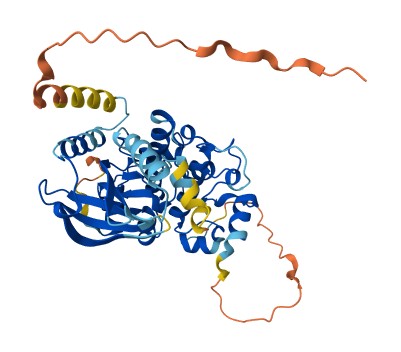

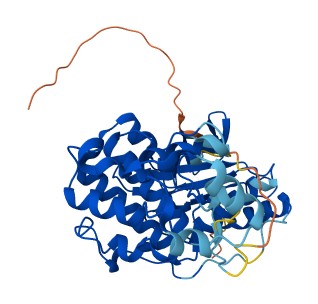

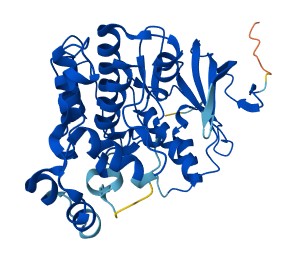

 **MAP2K2 MAPK3 MAPK1**


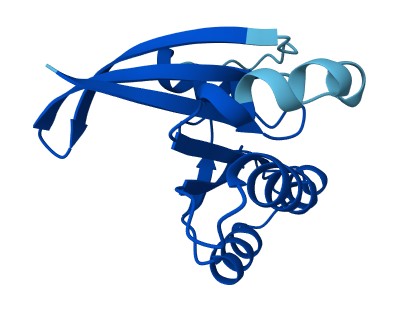

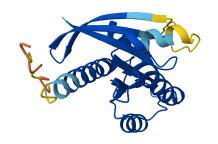

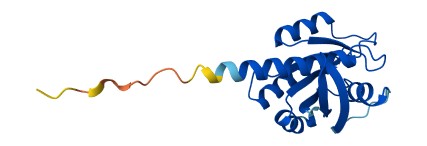

 **HRAS KRAS NRAS**


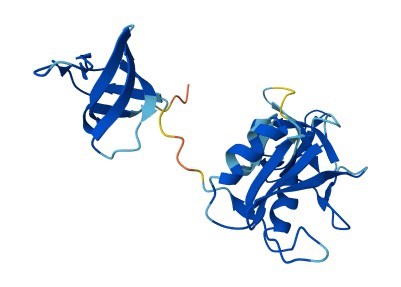

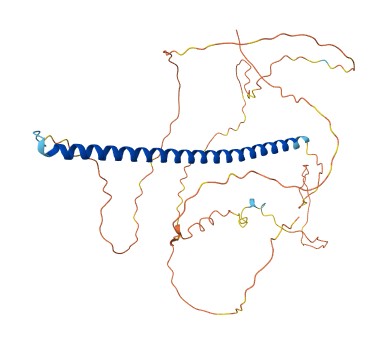

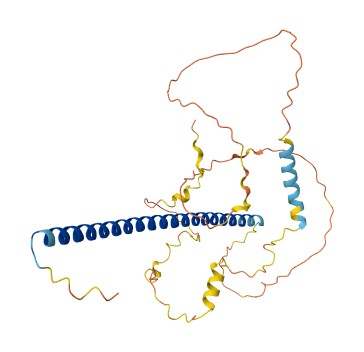

 **GRB2 FOS JUN**


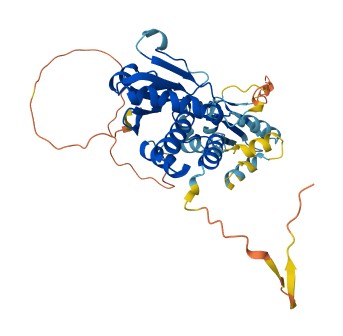

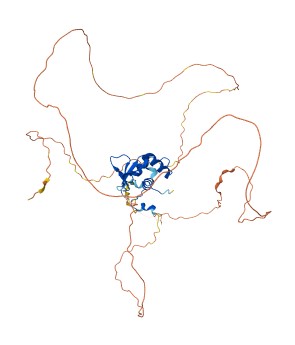

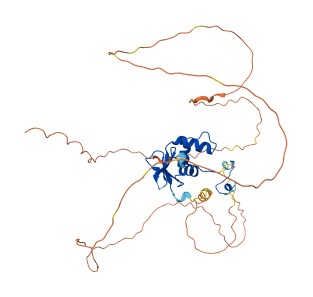

 **DUSP6 ELK1 ELK3**


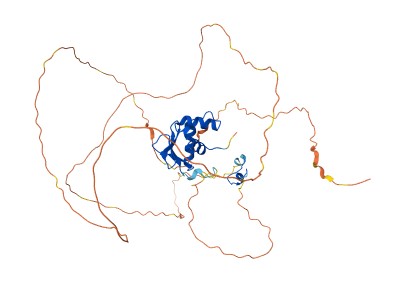

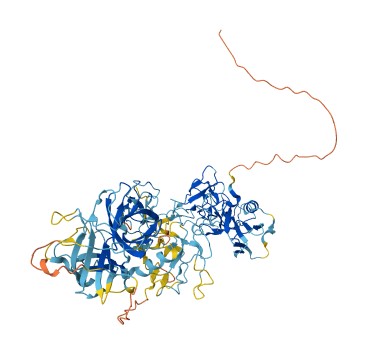

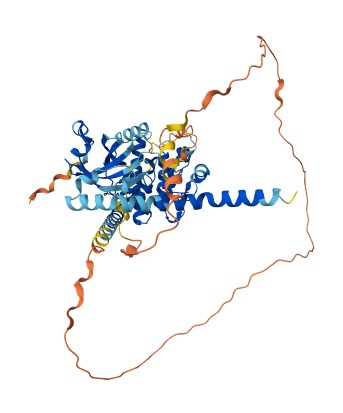

 **ELK4 MST1 STK3**


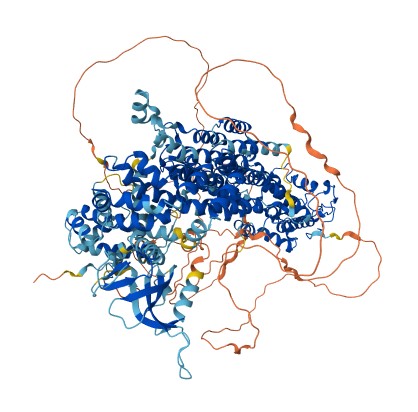

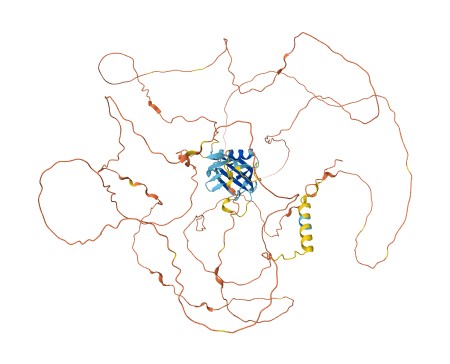

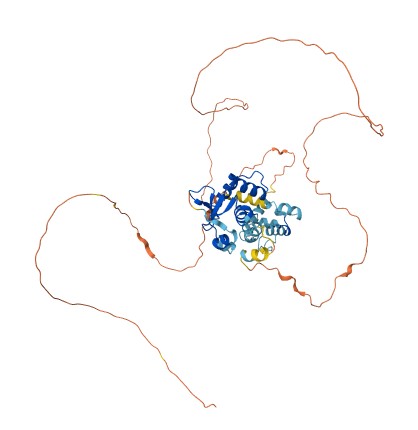

 **SOS1 GAB1 ETS1**

**Figure S2:** AlphaFold2-Based 3D Modeling of MAPK/ERK Pathway Genes in *B. bubalis.*
